# Tumor‐Associated Monocytes Reprogram CD8^+^ T Cells into Central Memory‐Like Cells with Potent Antitumor Effects

**DOI:** 10.1002/advs.202304501

**Published:** 2024-02-22

**Authors:** Zeliang Yang, Liang Liu, Zhenyu Zhu, Zixi Hu, Bowen Liu, Jingjing Gong, Yuan Jin, Juan Luo, Yichen Deng, Yan Jin, Guangxi Wang, Yuxin Yin

**Affiliations:** ^1^ Department of Pathology, Institute of Systems Biomedicine School of Basic Medical Sciences Beijing Key Laboratory of Tumor Systems Biology Peking University Health Science Center Beijing 100191 China; ^2^ Institute of Precision Medicine Peking University Shenzhen Hospital Shenzhen 518036 China; ^3^ Peking‐Tsinghua Center for Life Sciences Peking University Health Science Center Beijing 100191 China

**Keywords:** CD300LG, nitric oxide synthase, T cell exhaustion, T central memory‐like cells, tumor‐associated monocytes (TAMos)

## Abstract

CD8^+^ T cells are critical for host antitumor responses, whereas persistent antigenic stimulation and excessive inflammatory signals lead to T cell dysfunction or exhaustion. Increasing early memory T cells can improve T cell persistence and empower T cell‐mediated tumor eradication, especially for adoptive cancer immunotherapy. Here, it is reported that tumor‐associated monocytes (TAMos) are highly correlated with the accumulation of CD8^+^ memory T cells in human cancers. Further analysis identifies that TAMos selectively reprogram CD8^+^ T cells into T central memory‐like (T_CM_‐like) cells with enhanced recall responses. L‐NMMA, a pan nitric oxide synthase inhibitor, can mitigate TAMo‐mediated inhibition of T cell proliferation without affecting T_CM_‐like cell generation. Moreover, the modified T cells by TAMo exposure and L‐NMMA treatment exhibit long‐term persistence and elicit superior antitumor effects in vivo. Mechanistically, the transmembrane protein CD300LG is involved in TAMo‐mediated T_CM_‐like cell polarization in a cell‐cell contact‐dependent manner. Thus, the terminally differentiated TAMo subset (CD300LG^high^ACE^low^) mainly contributes to T_CM_‐like cell development. Taken together, these findings establish the significance of TAMos in boosting T‐cell antitumor immunity.

## Graphical abstract

Tumor‐associated monocytes (TAMos) reprogram activated CD8^+^ T cells to a central memory‐like phenotype with enhanced recall responses, which is mediated by the transmembrane protein CD300LG. CD8^+^ T cells cocultured with L‐NMMA‐treated TAMos exhibit long‐term persistence and functional fitness in the tumor microenvironment, thereby improving the efficacy of adoptive T cell therapy.

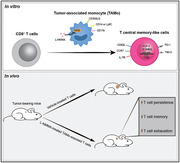

## 1 Introduction

Tremendous progress in adoptive T cell therapy (ACT) has been made for various hematological malignancies.^[^ ^1^ ^]^ However, ACT remains ineffective in the majority of solid tumors because of impaired function, insufficient activation, and poor persistence of tumor‐reactive T cells. Unlike the immune checkpoint blockade, ACT requires T cells to be activated and expanded in vitro to generate a sufficient number of functional T cells. Evidence to date indicates that differentiating T cells into a memory state during their activation or expansion in vitro could improve T cell persistence in vivo and potentiate their antitumor efficacy.^[^ ^2^, ^3^, ^4^ ^]^ Hence, optimizing T cell culture methods and identifying ways to sustain less differentiated T cells and improve the proportion of memory T (T_M_) cells appear to be critical for broadening the clinical applicability and efficacy of ACT.

When confronted with tumors, naïve T (T_Naïve_) cells mobilize to recognize and engage with pertinent antigens, subsequently undergoing activation and differentiation into diverse types of effector and memory cells that eradicate foreign antigens and provide long‐term immunity, respectively.^[^ ^5^ ^]^ Central memory T (T_CM_) cells are quiescent T_M_ cells that possess homeostatic proliferation capabilities and enhanced recall responses. T_CM_ cells exhibit elevated levels of lymphoid homing markers, including L‐selectin (CD62L) and C‐C chemokine receptor 7 (CCR7), as well as homeostatic cytokine receptors such as IL‐7 receptor (IL‐7R).^[^ ^6^ ^]^ T_CM_ cells are predominantly located in lymphoid organs, where they can be reactivated by antigen reencounters.^[^ ^7^ ^]^ Many transcription factors, such as TCF‐1, EOMES, ID3, RUNX1, and LEF1, have been found to support a central memory‐like program of T cells while providing persistent antitumor immunity.^[^ ^8^ ^]^ Depletion of BATF, inhibition of the mitochondrial pyruvate carrier (MPC), or suppression of CDK4/6 also promote T_CM_ cell generation.^[^ ^9^, ^10^, ^11^ ^]^ Nevertheless, the influence of multiple tumor‐associated myeloid cells on the state of T_CM_ cells remains largely unexplored.

The tumor microenvironment (TME) is characterized by hypoxia and the production of reactive molecules.^[^ ^12^ ^]^ Exogenous nitric oxide (NO), which is derived from multiple cellular sources including tumor cells, tumor‐associated monocytes (TAMos), tumor‐associated macrophages (TAMs), or fibroblasts, can impede T cell proliferation or precipitate the anergy and apoptosis of T cells.^[^ ^13^ ^]^ The targeting of nitric oxide synthase (NOS) has garnered considerable interest in clinical trials as a potential cancer treatment. However, different levels of NO can drive distinct molecular pathways and thus it can exhibit both antitumor or protumor functions. This makes it challenging to pursue an NOS‐targeting strategy in cancer patients.^[^ ^14^ ^]^ Another concern with targeting NOS is the possible off‐target consequences related to the application of pan NOS inhibitors, which may cause hypertension or impaired cardiac function.^[^ ^14^ ^]^ Therefore, further investigation is needed to ensure the accurate and safe utilization of NOS inhibitors as a potential NOS‐targeting cancer treatment.

Tumor burden can profoundly impact the immune cell differentiation trajectory and induce dozens of aberrant immune cells, such as tumor‐associated neutrophils (TANs), TAMos, TAMs, and erythroblast‐like cells (Ter‐cells).^[^ ^15^ ^]^ Despite the ability of these cells to facilitate tumor progression and immune evasion, some strategies which engage TANs to improve T cell immunotherapy have been described.^[^ ^16^, ^17^ ^]^ Thus, in certain circumstances, tumor‐associated cells may exhibit a beneficial effect. In the present study, we show that TAMos have the capacity to promote T_CM_‐like cell differentiation through direct cell‐cell contact during T cell activation or antigen rechallenge. At the transcriptomic and epigenetic levels, T cells cocultured with TAMos acquire a distinct landscape that is characterized by a constrained effector or exhaustion profile but enhanced central memory associated properties, resulting in robust tumor lytic ability in vitro. The utilization of an NOS inhibitor can mitigate the inhibition of T cell proliferation while preserving the beneficial effects of TAMos on T_CM_ cell generation. In a murine tumor model of ACT, the modified T cells induced by TAMo and NOS inhibitor treatment exhibit long‐term persistence and enhanced effector function, thus resulting in improved tumor control. Finally, we demonstrate that a terminally differentiated TAMo subset that highly expresses CD300LG is the chief contributor to the induction of T_CM_ cells. Together, our findings establish a novel role for TAMos in promoting the generation of T_CM_ cells, which in turn suggests an alternative way to improve the efficacy of ACT.

## 2 Results

### 2.1 Tumor‐Infiltrating CD14^+^ Monocytes are Positively Associated with the Accumulation of CD8^+^ Memory T Cells in Tumors

**Figure 1:**
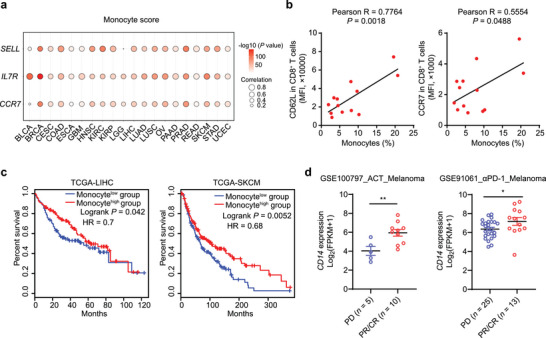
Tumor‐associated monocytes are related to T_M_ cell abundance. a) The correlation and *p* value between monocyte scores and *SELL*, *IL7R*, *CCR7* mRNA levels in different types of tumor tissues. b) PBMCs from lung cancer patients were stimulated with anti‐CD3 and anti‐CD28 antibodies plus rhIL‐2 for 96 h. The percentage of monocytes in untreated PBMCs and expression levels of the memory markers CD62L and CCR7 in CD8^+^ T cells from stimulated PBMCs were determined by flow cytometry, followed by Pearson's correlation test (*n* = 13). c) Overall survival curves of TCGA LIHC and SKCM patients grouped by mean expression values of monocyte signature genes (*n* (LIHC) = 364 patient samples, *n* (SKCM) = 458 patient samples). d)**,** *CD14* expression levels in tumor tissue samples from melanoma patients with PD, PR, or CR using RNA‐seq data from GSE100797_ACT_Melanoma or GSE91061_αPD‐1_Melanoma cohorts. Data are shown as means ± SEM d). Statistical significance was assessed using Pearson's correlation test a,b) a log‐rank (Mantel‐Cox) test c) or a two‐tailed unpaired Student's *t* test d). ^*^ *p* < 0.05, ^**^ *p* < 0.01.

The majority of T cells infiltrating the TME exhibit characteristics of exhausted T (T_EX_) cells. However, a small fraction still retains memory cell‐like properties.^[^ ^18^ ^]^ To determine whether TME‐derived cells exert a role in the production of T_M_ cells, we utilized spatial transcriptomic data^[^ ^19^ ^]^ to analyze the correlation between CD8^+^ T_M_ cells and a variety of myeloid cells in the TME. As shown in Figure S1a (Supporting Information), monocytes, rather than other myeloid cells such as neutrophils, macrophages, or dendritic cells (DCs), exhibited a strong colocalization with CD8^+^ T_M_ cells in the TME. To further confirm this finding, we calculated monocyte scores to infer the percentages of monocytes in 20 types of tumors from The Cancer Genome Atlas (TCGA). As shown in **Figure** **1**a, monocyte scores showed a significant positive correlation with the expression of T_M_ cell marker genes including *SELL*, *CCR7*, and *IL7R* in most tumor types. To further explore this association during T cell activation in vitro, we stimulated peripheral blood mononuclear cells (PBMCs) derived from lung cancer patients with anti‐CD3 and anti‐CD28 antibodies and observed that levels of CD62L (encoded by *SELL*) and CCR7 expression in activated CD8^+^ T cells were positively correlated with the proportion of monocytes (Figure 1b; Figure S1b,c, Supporting Information).

Given that T_M_ cell enrichment is associated with improved clinical outcomes in cancer patients,^[^ ^20^ ^]^ we therefore explored the prognostic value of monocytes. Indeed, higher levels of monocyte signature genes were associated with longer overall survival rates in liver hepatocellular carcinoma (LIHC) and skin cutaneous melanoma (SKCM) patients (Figure 1c). In addition, we also analyzed the transcriptomes of tumor tissues and compared them with clinical responses in cohorts of melanoma patients receiving ACT or anti‐PD‐1 therapy.^[^ ^21^, ^22^ ^]^ As shown in Figure 1d, expression of the monocyte signature gene *CD14* was elevated in the patient groups exhibiting partial response (PR) or complete response (CR) relative to the group of patients with progressive disease (PD). These data suggest that TAMos may play a beneficial role in T_M_ cell generation and host antitumor immunity.

### 2.2 Tumor‐Associated Monocytes Selectively Promote the Central Memory‐Like T Cell Polarization

**Figure 2:**
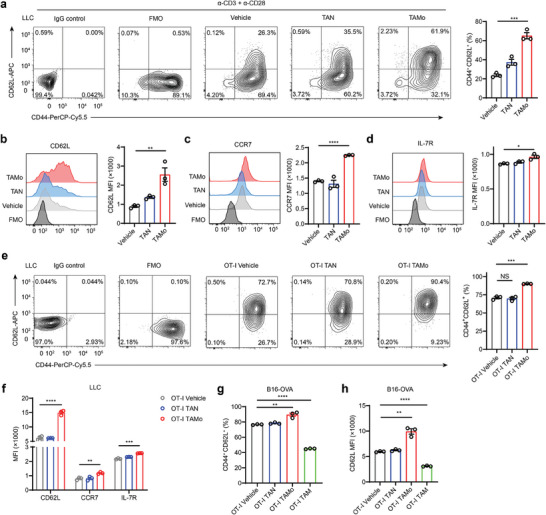
TAMos derived from the spleens of tumor‐bearing mice promote T_CM_ cell generation. a) CD8^+^ T cells were cocultured with TAMos or TANs from the spleens of LLC tumor‐bearing mice (four pooled mice) and stimulated with anti‐CD3 and anti‐CD28 antibodies (α‐CD3 + α‐CD28), followed by flow cytometry to determine the proportion of CD44^+^CD62L^+^ cells in the activated CD8^+^ T cells (*n* = 3 cell cultures). FMO, fluorescence minus one. b–d) Expression levels of CD62L b), CCR7 c), and IL‐7R d) in CD8^+^ T cells cocultured with TAMos or TANs in the presence of anti‐CD3 and anti‐CD28 antibodies (*n* = 3 cell cultures). FMO, fluorescence minus one. e) OT‐I cells were cocultured with TAMos or TANs sorted from the spleens of LLC tumor‐bearing mice (six pooled mice) under OVA_257‐264_ stimulation, and the percentages of CD44^+^CD62L^+^ cells were determined by flow cytometry (*n* = 3 cell cultures). FMO, fluorescence minus one. f) CD62L, CCR7, and IL‐7R expression levels in OT‐I CD8^+^ T cells cocultured with TAMos or TANs in the presence of OVA_257‐264_ peptides (*n* = 3 cell cultures). g,h) TANs, TAMos, or TAMs were sorted from B16‐OVA tumor‐bearing mice (ten pooled mice) and cocultured with OT‐I cells under OVA_257‐264_ stimulation. CD44^+^CD62L^+^ cell proportions (g, *n* = 3 cell cultures) and CD62L levels (h, *n* = 3 cell cultures) in CD8^+^ T cells were determined by flow cytometry. Data are representative of three independent experiments and shown as means ± SEM. Statistical significance was assessed using a two‐tailed unpaired Student's *t* test. ^*^ *p* < 0.05, ^**^ *p* < 0.01, ^***^ *p* < 0.001, and ^****^ *p* < 0.0001. NS, not significant.

To determine whether TAMos have the potential to directly affect T_M_ cell differentiation, we sorted TAMos and TANs from spleens of LLC tumor‐bearing mice (Figure S2a, Supporting Information) and cocultured them with CD8^+^ T cells under activation of anti‐CD3 and anti‐CD28 antibodies. We then determined the proportion of T_M_ cells using flow cytometry. Unlike vehicle or TANs, TAMos substantially increased the proportion of T_CM_ cells (designated as CD44^+^CD62L^+^ T cells) after T cell priming and activation (**Figure** **2**a). We also noticed that T cells cocultured with TAMos exhibited higher expression levels of membrane molecules including CD62L, CCR7, and IL‐7R (Figure 2b–d), which are essential for the maintenance of the central memory phenotype.^[^ ^6^ ^]^ These data indicate that TAMos induce T_CM_ cell generation during T cell activation. To obtain corroborating evidence, we employed OT‐I cells expressing a transgenic T cell receptor (TCR) which is specific for OVA_257‐264_ peptides. As shown in Figure 2e,f, TAMos derived from spleens of LLC tumor‐bearing mice markedly enhanced T_CM_ cell differentiation as indicated by elevated percentages of CD44^+^CD62L^+^ T cells and increased levels of CD62L, CCR7, and IL‐7R expression during T cell antigen‐specific activation compared with vehicle or TANs. Similar observations were made using TAMos sorted from the spleens of B16‐OVA tumor‐bearing mice (Figure 2g,h; Figure S2b, Supporting Information). In contrast, TAMs inhibited the formation of T_CM_ cells and downregulated CD62L levels (Figure 2g,h). These findings indicate that TAMos act as a distinct monocytic subset to promote T_CM_ cell generation under T cell antigen‐specific priming.

Given that TAMos also extensively infiltrate tumors in addition to peripheral immune organs,^[^ ^23^ ^]^ we further explored the effect of TAMos derived from the TME on T cell differentiation. Consistent with our findings using splenic TAMos, TME‐derived TAMos robustly induced T_CM_ cell differentiation and elevated CD62L levels in activated T cells (Figure S2c,d, Supporting Information). Collectively, these results demonstrate that TAMos derived from tumor‐bearing mice have the capacity to induce T_CM_ cell generation during T cell activation.

### 2.3 T_CM_‐Like Cells Induced by TAMos are Distinct from Effector T Cells and Exhausted T Cells

**Figure 3:**
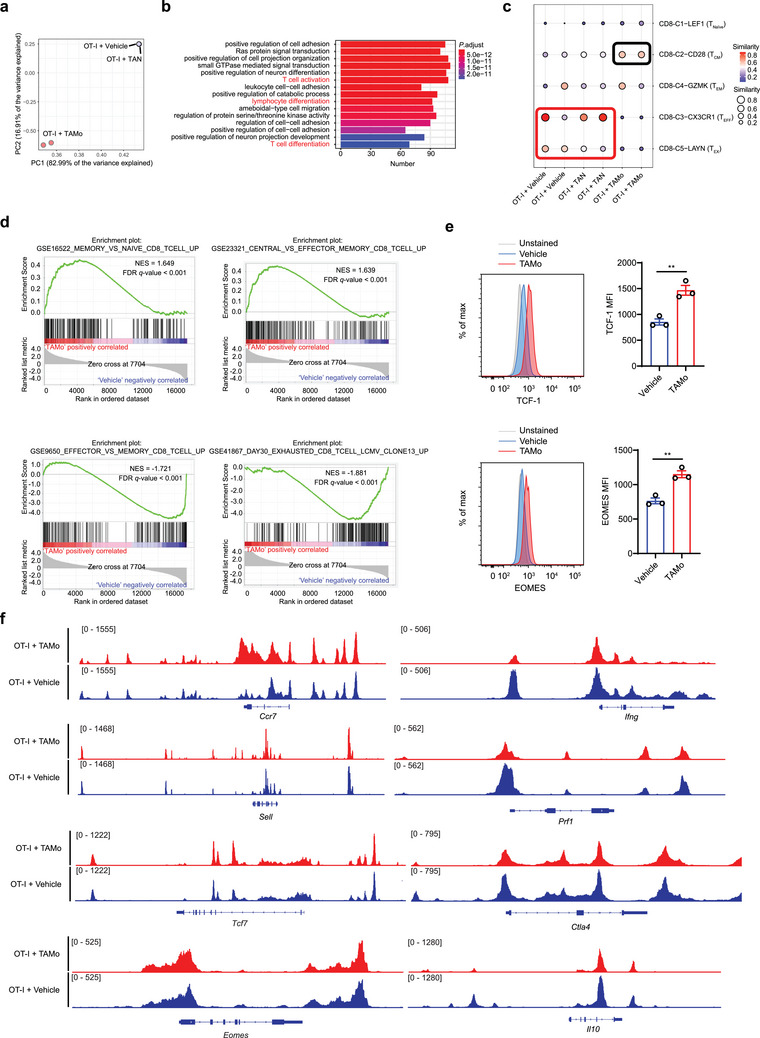
TAMos reprogram the T cell landscape during T cell activation. RNA‐seq analysis a–d) of CD8^+^ T cells purified from coculture systems in which murine OT‐I cells were exposed to vehicle, TAMos, or TANs sorted from the spleens of LLC tumor‐bearing mice and stimulated with OVA_257‐264_ peptides (*n* = 2 cell cultures). a) Principal components analysis of transcriptomes of CD8^+^ T cells under different conditions. b) Enrichment analysis of upregulated genes in T cells cocultured with TAMos compared with T cells cultured alone using GO datasets. Pathways related to T cell biological processes are highlighted in red. c) Bubble map displaying the similarities among vehicle, TAN, or TAMo‐treated murine T cells and different T cell subsets isolated from the PBMCs and tumor tissues of human NSCLC patients. T_Naïve_, naïve T cells; T_CM_, central memory T cells; T_EM_, effector memory T cells; T_EFF_, effector T cells; T_EX_, exhausted T cells. d) GSEA plots showing representative pathways enriched in murine CD8^+^ T cells cocultured with TAMos or cultured alone. NES, normalized enrichment score. e) CD8^+^ T cells were cultured alone or cocultured with TAMos derived from the spleens of LLC tumor‐bearing mice (five pooled mice) and stimulated with anti‐CD3 and anti‐CD28 antibodies for 48 h, followed by flow cytometry to determine TCF‐1 and EOMES protein levels. Unstained activated CD8^+^ T cells were used as a negative control (*n* = 3 cell cultures). f) IGV plots of ATAC‐seq peaks at *Ccr7*, *Sell*, *Tcf7*, *Eomes*, *Ifng*, *Prf1*, *Ctla4*, or *Il10* loci in OT‐I CD8^+^ T cells cultured alone or cocultured with TAMos derived from the spleens of LLC tumor‐bearing mice. Data are representative of three independent experiments e) and shown as means ± SEM. Statistical significance was assessed using a two‐tailed unpaired Student's *t* test e). ^**^ *p* < 0.01.

To systematically characterize the effects of TAMos on the T cell transcriptional landscape, we performed RNA‐sequencing (RNA‐seq) analysis of activated CD8^+^ T cells sorted from cocultures of OT‐I T cells together with vehicle, TANs, or TAMos. Unlike TANs, which only slightly affected T cell expression profiles, coculture with TAMos substantially reprogrammed the T cell transcriptome (**Figure** **3**a). Gene Ontology (GO) analysis showed that genes upregulated in TAMo‐cocultured T cells were enriched in T cell activation and differentiation‐associated pathways (Figure 3b), indicating that TAMos affect T cell differentiation programs. On the other hand, the downregulated genes in TAMo‐cocultured T cells were mainly associated with processes related to cell division (Figure S3a, Supporting Information), in agreement with previous reports that TAMos from tumor‐bearing mice suppress T cell proliferation.^[^ ^24^ ^]^

To further clarify the effects of TAMos on T cell differentiation programs, we constructed a logistic regression model using single‐cell transcriptomic data from different subtypes of CD8^+^ T cells derived from non‐small cell lung cancer (NSCLC) patients.^[^ ^25^ ^]^ As shown in Figure 3c, transcriptomes of T cells cocultured with TAMos more closely resembled the patterns of T_CM_ cells compared with vehicle‐treated or TAN‐exposed T cells. In the latter groups, the CD8^+^ T cells exhibited more similarity to effector T (T_EFF_) and T_EX_ cells. We also integrated our bulk RNA‐seq data with the transcriptomes of conventional T_CM_, effector memory T (T_EM_), and T_Naïve_ cells, followed by principal components analysis (PCA). As shown in Figure S3b (Supporting Information), TAMo‐cocultured T cells were more similar to conventional T_CM_ cells than T_EM_ and T_Naïve_ cells, an observation which further supports the conclusion that TAMos skew CD8^+^ T cell differentiation to a central memory‐like phenotype. Furthermore, gene set enrichment analysis (GSEA)^[^ ^26^ ^]^ revealed that TAMos triggered T cells to upregulate genes associated with memory (especially central memory) CD8^+^ T cells but also, to downregulate genes associated with T cell effector function or exhaustion state (Figure 3d). Consistent with these observations, TAMo‐cocultured T cells exhibited higher expression levels of *Ccr7*, *Il7r*, and *Sell*, and several transcription factors such as *Tcf7*, *Lef1*, and *Runx1*, which promote the generation and persistence of T_CM_ cells (Figure S3c, Supporting Information). In contrast, T cells cultured alone or cocultured with TANs upregulated expression of genes encoding effector factors, including *Ifng, Tnf*, and *Prf1*, and immune checkpoints, such as *Il10*, *Pdcd1*, *Havcr2*, and *Ctla4* (Figure S3c, Supporting Information). These results were confirmed by RT‐qPCR analysis (Figure S3d, Supporting Information). Considering the critical role of transcription factors such as TCF‐1 and EOMES in T_CM_ cell differentiation,^[^ ^27^ ^]^ we also found that coculture with TAMos elevated levels of TCF‐1 and EOMES proteins in activated T cells as determined by flow cytometry (Figure 3e). Together, these data indicate that TAMos drive T cell transcriptional profiles toward a central memory‐like status, and away from an effector or exhaustion program.

To better understand the regulatory effects of TAMos on T cell transcription, we performed an assay for transposase‐accessible chromatin with high‐throughput sequencing (ATAC‐seq) to identify the accessible chromatin regions of T cells. Compared with control, coculture of OT‐I cells with TAMos increased the chromatin accessibility of genes related to T cell differentiation and memory status, including *Ccr7*, *Sell*, *Tcf7*, and *Eomes*, whereas the chromatin accessibility at the *Ifng*, *Prf1*, *Ctla4*, and *Il10* loci was decreased (Figure 3f; Figure S3e, Supporting Information). Collectively, our data demonstrate that TAMos reprogram T cells to acquire a distinct epigenetic and transcriptional landscape with a less differentiated state in the process of T cell activation.

### 2.4 Human TAMos Elicit Similar Effects on T_CM_‐Like Cell Polarization Upon Antigen Rechallenge

**Figure 4:**
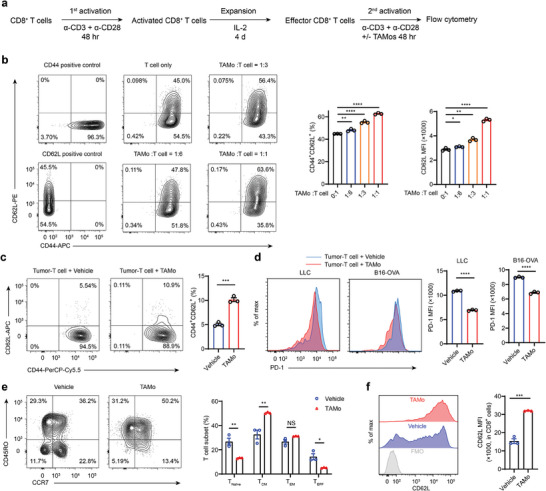
TAMos potentiate the T cell memory phenotype and attenuate the T cell exhaustion state after antigen rechallenge. a) Flow diagram illustrating the protocol used for antigen rechallenge experiments. b) Expanded CD8^+^ T cells were cocultured with TAMos derived from the spleens of LLC tumor‐bearing mice at different ratios and reactivated with anti‐CD3 and anti‐CD28 antibodies for 48 h, followed by flow cytometry to determine the percentages of CD44^+^CD62L^+^ cells and CD62L levels (four pooled mice, *n* = 3 cell cultures). c) CD8^+^ T cells were sorted from the TME of LLC tumor‐bearing mice and cocultured with autogenous TAMos in the presence of anti‐CD3 and anti‐CD28 antibodies plus IL‐2 for 48 h, followed by flow cytometry to determine the percentages of CD44^+^CD62L^+^ cells in CD8^+^ T cells (eight pooled mice, *n* = 3 cell cultures). d) Expression levels of PD‐1 in CD8^+^ T cells sorted from the TME of LLC or B16‐OVA tumor‐bearing mice and cocultured with TAMos after anti‐CD3 and anti‐CD28 plus IL‐2 stimulation for 48 h (eight pooled LLC tumor‐bearing mice or six pooled B16‐OVA tumor‐bearing mice, *n* = 3 cell cultures). e,f) Human CD8^+^ T cells and TAMos were sorted from PBMCs of lung cancer patients (*n* = 7 pooled samples). CD8^+^ T cells were cultured alone or cocultured with the sorted TAMos and activated with anti‐CD3 and anti‐CD28 antibodies plus rhIL‐2 for 96 h, followed by flow cytometry to determine the proportions of different T cell subsets (e, T_Naïve_, CCR7^+^CD45RO^−^; T_CM_, CCR7^+^CD45RO^+^; T_EM_, CCR7^−^CD45RO^+^; T_EFF_, CCR7^−^CD45RO^−^; *n* = 3 cell cultures) and CD62L expression levels in CD8^+^ T cells (f, *n* = 3 cell cultures). FMO, fluorescence minus one. Data are representative of two independent experiments and shown as means ± SEM b–f). Statistical significance was assessed using a two‐tailed unpaired Student's *t* test b–f). ^*^ *p* < 0.05, ^**^ *p* < 0.01, ^***^ *p* < 0.001, and ^****^ *p* < 0.0001. NS, not significant.

To evaluate the potential value of TAMos in ACT in which reactivated T cells in vitro are transferred into tumor‐bearing hosts,^[^ ^28^, ^29^ ^]^ we assessed the effects of TAMos on the differentiation of activated T cells upon antigen rechallenge. CD8^+^ T cells were primed with anti‐CD3 and anti‐CD28 antibodies for 2 days and then expanded with IL‐2 for a further 4 days. These CD8^+^ T cells were then reactivated with or without TAMos sorted from LLC tumor‐bearing mice (**Figure** **4**a). As shown in Figure 4b, TAMos reshaped reactivated T cells into a central memory‐like phenotype in a dose‐dependent manner with increased proportions of CD44^+^CD62L^+^ cells and elevated CD62L levels. We also purified tumor‐infiltrating CD8^+^ T cells and cocultured them with TAMos in situ upon reactivation. Flow cytometry analysis demonstrated that the percentage of T_CM_ cells was substantially increased in the presence of TAMos (Figure 4c). Because reactivated T cells are prone to acquire an exhaustion phenotype,^[^ ^9^ ^]^ we also estimated the expression levels of the exhaustion marker PD‐1. Flow cytometry analysis showed that PD‐1 levels were downregulated in the reactivated T cells cocultured with autologous TAMos derived from either the LLC or B16‐OVA tumor‐bearing mice models (Figure 4d). Collectively, these data indicate that TAMos from tumor‐bearing mice also promote T_CM_ cell generation with a lower exhaustion state upon T cell reactivation.

Our studies with the mice models prompted us to investigate the role of TAMos in human CD8^+^ T cell reactivation responses. Thus, we sorted TAMos and CD8^+^ T cells from pooled PBMCs of lung cancer patients and reactivated CD8^+^ T cells in the presence or absence of TAMos. As expected, coculture with TAMos robustly induced a phenotypic shift of CD8^+^ T cells to T_CM_‐like cells, manifested as an increased proportion of T_CM_ (CD45RO^+^CCR7^+^) cells and decreased proportion of T_Naïve_ (CD45RO^−^CCR7^+^) or T_EFF_ (CD45RO^−^CCR7^−^) cells (Figure 4e). Furthermore, CD8^+^ T cells cocultured with TAMos expressed higher levels of CD62L in comparison to vehicle‐treated T cells (Figure 4f). Thus, our data demonstrate that TAMos in cancer patients also have the capacity to enhance CD8^+^ T_CM_‐like cell polarization.

### 2.5 L‐NMMA Treated TAMos Enhance T_CM_ Cell Differentiation without Affecting T Cell Proliferation

**Figure 5:**
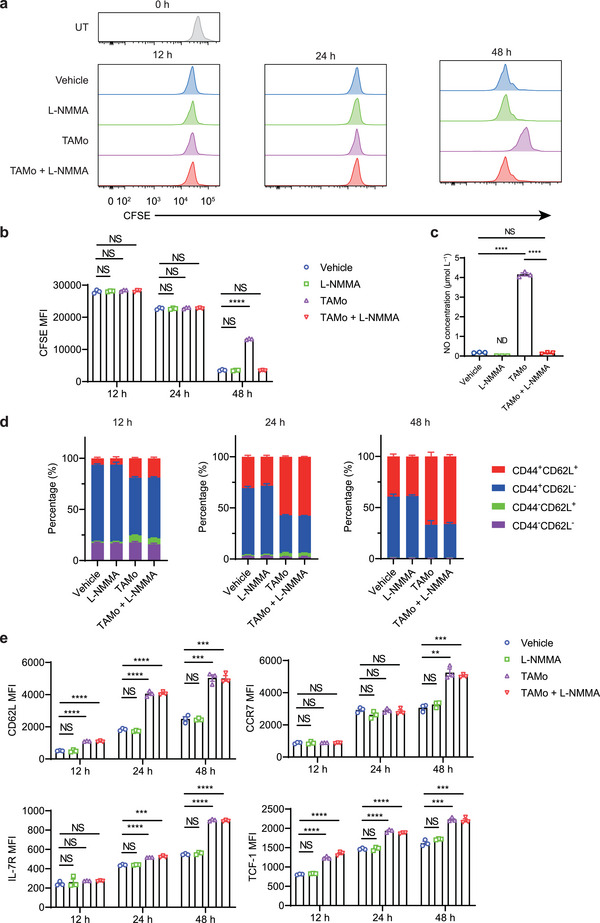
TAMos promote T_CM_ cell differentiation independent of NOS2‐mediated inhibition of T cell proliferation. a,b) CFSE‐labeled CD8^+^ T cells were cultured alone or cocultured with TAMos derived from the spleens of LLC tumor‐bearing mice (ten pooled mice) in the presence or absence of L‐NMMA under stimulation of anti‐CD3 and anti‐CD28 antibodies, and T cell proliferation was determined by mean fluorescence intensity of CFSE at 12, 24, or 48 h post T cell activation (*n* = 3 cell cultures). c) NO concentrations in supernatants of CD8^+^ T cell activated with anti‐CD3 and anti‐CD28 antibodies alone (Vehicle), in the presence of L‐NMMA, in the presence of TAMos or in the presence of TAMos and L‐NMMA (*n* = 3 cell cultures). ND, not detected. d,e) Flow cytometry analysis of the proportions of different T cell subsets (d, *n* = 3 cell cultures) and the expression levels of CD62L, CCR7, IL‐7R, and TCF‐1 (e, *n* = 3 cell cultures) in activated CD8^+^ T cells cultured alone or cocultured with TAMos sorted from LLC tumor‐bearing mice (ten pooled mice) for 12, 24, or 48 h with or without L‐NMMA. Data are shown as means ± SEM. Statistical significance was assessed using a two‐tailed unpaired Student's *t* test. ^**^ *p* < 0.01, ^***^ *p* < 0.001, and ^****^ *p* < 0.0001. NS, not significant.

Previous reports have suggested that TAMos suppress T cell proliferation via elevated NOS2 expression and NO secretion.^[^ ^24^, ^30^ ^]^ We therefore sought to exclude the potential effects of NO on T cell expansion by employing a pan NOS inhibitor, L‐NMMA, in our experiments. Using the CFSE dilution assay, we observed that TAMos primarily inhibited T cell proliferation in the later stages of T cell activation (48 h post‐activation) (**Figure** **5**a,b). The addition of L‐NMMA significantly reduced NO production in the cocultures (Figure 5c). Importantly, when L‐NMMA was added, TAMos could expand activated CD8^+^ T cells to an extent similar to that of the vehicle control (Figure 5a,b). We then assessed the effects of TAMos on the T cell memory state at multiple time points during T cell activation in different culture conditions. As shown in Figure 5d,e, TAMos promoted the generation of the central memory phenotype within the first 24 h post T cell activation, as evident by increased percentages of CD44^+^CD62L^+^ T cells and upregulated expression of CD62L, IL‐7R, and TCF‐1 in the activated T cells. Forty‐eight hours later, the central memory phenotype of TAMo‐cocultured T cells was further amplified compared to vehicle‐treated T cells. Moreover, CD8^+^ T cells activated in the presence of TAMos and L‐NMMA also displayed higher proportions of T_CM_ cells and increased levels of CD62L, CCR7, IL‐7R, and TCF‐1 proteins at multiple time points, similar to CD8^+^ T cells cocultured with TAMos alone (Figure 5d,e). In contrast, treatment with L‐NMMA alone had no effect on T cell expansion or differentiation (Figure 5a,b,d,e). These results demonstrate that TAMos can promote T_CM_ cell production that is independent of NO production and inhibition of T cell proliferation.

To further substantiate this conclusion, we performed knockdown experiments to silence endogenous NOS2 in TAMos, thereby inhibiting NO secretion and restoring T cell proliferation (Figure S4a–c, Supporting Information). We then cocultured control or NOS2 knockdown TAMos with CD8^+^ T cells upon T cell activation. Flow cytometry analysis showed that downregulation of NOS2 expression did not influence the differentiation of T_CM_ cells mediated by TAMos (Figure S4d,e, Supporting Information), further confirming that the effects of TAMos on T cell proliferation and memory state are uncoupled. We also determined the activation state of T cells cocultured with L‐NMMA‐treated TAMos. As shown in Figure S4f (Supporting Information), T cells cocultured with L‐NMMA‐treated TAMos showed equivalent T cell activation signaling compared with vehicle‐treated T cells. Thus, our results indicate that L‐NMMA‐treated TAMos promote T_CM_ cell generation without affecting T cell activation and expansion.

### 2.6 T Cells Exposed to TAMos and L‐NMMA In Vitro Exhibit Potent Antitumor Effects In Vivo

**Figure 6:**
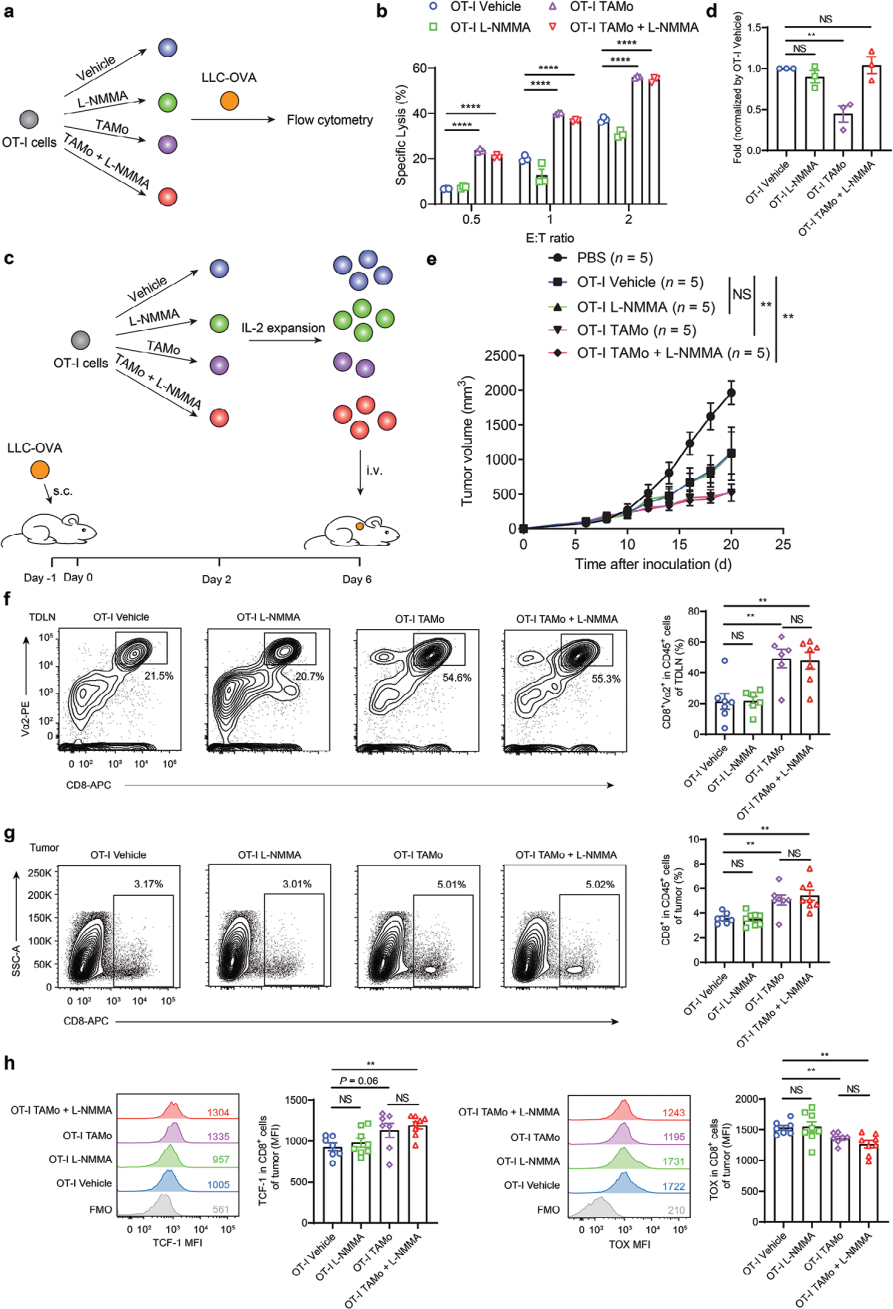
T cells cocultured with TAMos exhibited enhanced antitumor activity in vitro and in vivo independent of NO signaling. a) Diagram illustrating protocol for T cell killing assay. b) OT‐I cells were cultured alone or cocultured with TAMos derived from the spleens of LLC tumor‐bearing mice (ten pooled mice) and activated with OVA_257‐264_ peptides for 48 h in the presence or absence of L‐NMMA, followed by incubation with LLC‐OVA tumor cells. The percentages of specific lysis were determined by flow cytometry (*n* = 3 cell cultures). E refers to effector cells (OT‐I cells); T refers to target cells (LLC‐OVA tumor cells). c) Flow diagram illustrating the adoptive T cell transfer model. d–h) OT‐I cells were cultured alone or cocultured with TAMos derived from the spleens of LLC tumor‐bearing mice (twenty pooled mice) in the presence or absence of L‐NMMA upon OVA_257‐264_ stimulation for 48 h. After expansion for 4 days using IL‐2, those T cells were transferred into NOD‐SCID mice bearing LLC‐OVA tumor. d) Fold changes in the number of T cells in different groups after IL‐2 expansion normalized by the vehicle‐treated T cell group (*n* = 3 replicates). e) Tumor volumes in the different groups of mice were monitored every two days (*n* = 5 mice per group). f,g) On day 4 after T cell transfer, the proportions of CD8^+^ T cells in TDLNs (f, *n* = 6 or 7 mice per group) and tumors (g, *n* = 7 or 8 mice per group) were determined by flow cytometry. h) Flow cytometry analysis showing the expression levels of TCF‐1 and TOX in transferred CD8^+^ T cells infiltrating tumors (*n* = 7 or 8 mice per group). FMO, fluorescence minus one. Data are shown as means ± SEM. Statistical significance was assessed using a two‐tailed unpaired Student's *t* test b,d,f–h) or two‐way ANOVA e). ^**^ *p* < 0.01, ^****^ *p* < 0.0001. NS, not significant.

T_CM_ cells are typically characterized by enhanced recall responses and antitumor activity.^[^ ^9^ ^]^ Therefore, we performed T cell killing assays to determine the antitumor activity of TAMo‐induced T_CM_ cells (**Figure** **6**a). Compared with control T cells, T cells cocultured with TAMos in the presence or absence of L‐NMMA exhibited increased cytotoxicity against LLC‐OVA tumor cells (Figure 6b). However, treatment with L‐NMMA alone did not enhance T cell antitumor activity (Figure 6b). These results demonstrate that TAMos can improve T cell‐mediated cytotoxicity in vitro independent of NOS2 activity. To further evaluate the therapeutic potential of TAMos in ACT, we cultured OT‐I cells alone or cocultured OT‐I cells with TAMos in the presence or absence of L‐NMMA upon T cell activation for 2 days. After expanding T cells with IL‐2 for 4 days, we transferred them into NOD‐SCID mice bearing LLC‐OVA tumors (Figure 6c). We first assessed the T cell memory status after IL‐2 expansion. As shown in Figure S5a,b (Supporting Information), T cells pre‐exposed to TAMos with or without L‐NMMA still exhibited an elevated proportion of T_CM_ cells and increased CD62L expression levels after IL‐2 treatment. Importantly, although TAMo‐cocultured T cells exhibited a lower expansion rate, T cells cocultured with L‐NMMA‐exposed TAMos and vehicle‐treated T cells proliferated to a similar extent (Figure 6d). After T cell transfer into NOD‐SCID mice bearing LLC‐OVA tumors, tumor volumes were measured over time. As shown in Figure 6e, T cells pre‐exposed to TAMos with or without L‐NMMA significantly inhibited tumor growth. Moreover, mice receiving T cells treated with L‐NMMA alone or vehicle‐treated T cells exhibited similar tumor growth curves (Figure 6e). These data indicate that TAMo exposure significantly enhances T cell antitumor activity independent of NO signaling.

To elucidate the mechanisms underlying T cell‐mediated inhibition of tumor growth in vivo following TAMo coculture, we collected tumor‐draining lymph nodes (TDLNs) and tumors from tumor‐bearing mice on day 4 after T cell transfer. Flow cytometry analysis revealed that mice receiving TAMo‐cocultured T cells, with or without L‐NMMA, displayed higher percentages of CD8^+^ T cells in their TDLNs and tumors compared to mice receiving vehicle or L‐NMMA‐treated T cells (Figure 6f,g). This finding demonstrates that TAMos confer long‐term persistence to T cells in vivo independent of NO production. We also used flow cytometry to analyze the expression levels of the memory‐associated marker TCF‐1 and the exhaustion‐related marker TOX in CD8^+^ T cells infiltrating the tumors. Compared to mice receiving vehicle‐treated T cells, tumor infiltrating T cells from mice receiving TAMo‐cocultured T cells exhibited higher levels of TCF‐1 and lower levels of TOX (Figure 6h). Of note, L‐NMMA treatment had no influence on the TAMo‐induced memory‐like phenotype in vivo (Figure 6h). We also evaluated the production of effector cytokines in the transferred T cells in various tissues of mice receiving vehicle‐treated T cells or TAMo‐cocultured T cells treated with L‐NMMA. As shown in Figure S5c (Supporting Information), levels of IFN‐γ and TNF in transferred T cells in the TDLNs, spleens, and PBMCs of mice receiving T cells pre‐exposed to L‐NMMA‐treated TAMos were greater than the transferred T cells in tissues from mice receiving T cells exposed only to the vehicle. Together, these data support the conclusion that CD8^+^ T cells after TAMo exposure acquire superior and long‐term antitumor efficacy in vivo.

### 2.7 CD300LG is Involved in TAMo‐Mediated T_CM_‐Like Cell Polarization

**Figure 7:**
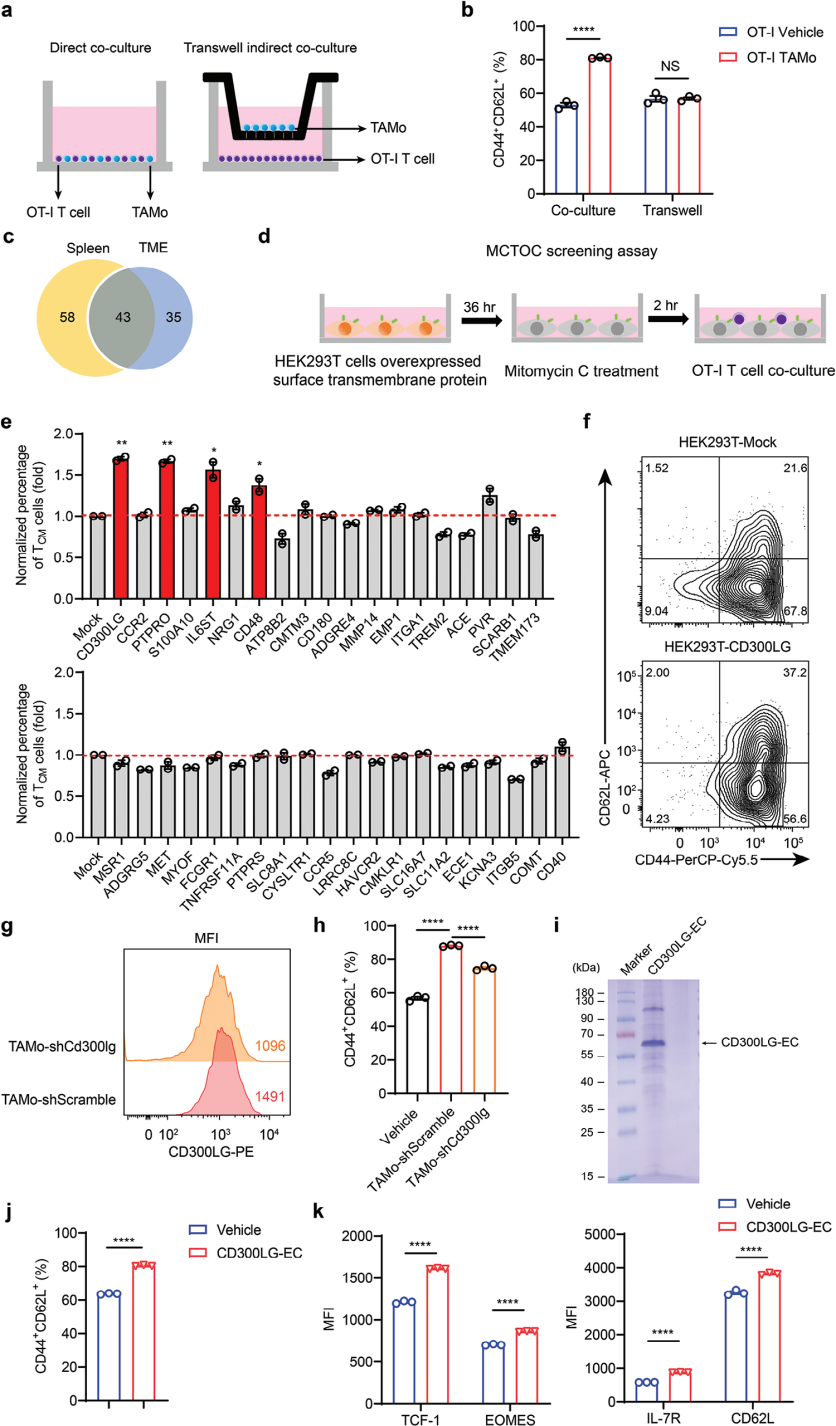
CD300LG upregulated in TAMos is associated with T_CM_ cell differentiation. a) Schematic representation of the direct coculture and Transwell indirect coculture protocols with OT‐I T cells and TAMos. b) OT‐I T cells were cocultured with TAMos sorted from the spleens of LLC tumor‐bearing mice (nine pooled mice) together or in a Transwell system for 48 h, followed by flow cytometry to determine percentages of CD44^+^CD62L^+^ cells in CD8^+^ T cells (*n* = 3 cell cultures). c) Venn diagram denoting the overlap of genes encoding the top upregulated surface transmembrane proteins in TAMos (fold change > 10, adjusted *p* value < 0.05 and FPKM > 0.5) from the spleens and the TME of tumor‐bearing mice. d) Schematic plot of the MCTOC screening assay. e) Flow cytometry analysis of fold changes of T_CM_ cell percentages in CD8^+^ T cells from coculture of OT‐I T cells and mitomycin C‐treated HEK293T cells expressing mCherry‐tagged mock or surface transmembrane proteins (*n* = 2 cell cultures). f) The proportion of T_CM_ cells in CD8^+^ T cells from coculture of OT‐I T cells and mitomycin C‐treated HEK293T cells expressing mCherry‐tagged mock or CD300LG protein. g) Flow cytometry analysis showing CD300LG levels in TAMos expressing shRNA targeting *Cd300lg* or scramble shRNA. h) OT‐I T cells were cocultured with TAMos infected with lentivirus encoding shRNA targeting *Cd300lg* or scramble shRNA during T cell activation, followed by flow cytometry to determine proportion of T_CM_ cells. i) Coomassie‐stained SDS‐polyacrylamide gel of recombinant CD300LG‐EC. j,k) OT‐I cells were cultured in the presence of recombinant CD300LG‐EC for 48 h during T cell activation, followed by flow cytometry to determine percentages of CD44^+^CD62L^+^ cells (j, *n* = 3 cell cultures) and the expression levels of TCF‐1, EOMES, IL‐7R, and CD62L (k, *n* = 3 cell cultures). Data are representative of two independent experiments b,e–h) and shown as means ± SEM b,e,h,j,k). Statistical significance was assessed using a two‐tailed unpaired Student's *t* test b,e,h,j,k). NS, not significant, ^*^ *p* < 0.05, ^**^ *p* < 0.01, ^****^ *p* < 0.0001.

To elucidate the mechanisms by which TAMos promote T_CM_ cell differentiation, we first examined whether or not direct cell‐cell contact was required. In contrast to our results using a direct coculture system, the separation of TAMos and OT‐I T cells using a Transwell system completely abrogated the ability of TAMos to induce T_CM_ cell polarization, indicating the involvement of a mechanism dependent on cell‐cell contact (**Figure** **7**a,b). Because the most common way to carry out biological functions through direct cell‐cell contact is via the interaction of cell surface transmembrane proteins, we then explored whether transmembrane proteins specifically expressed in TAMos could induce T_CM_ cell differentiation. To this end, we performed RNA‐seq analysis on TAMos and TANs from the spleens and the TME of tumor‐bearing mice. Differentially expressed gene (DEG) analysis revealed that 5039 and 4146 genes (such as *Ly6c2*, *Fn1*, and *S100a10*) were upregulated in TAMos derived from the spleens and the TME, respectively (Figure S6a,b, Supporting Information). In contrast, TANs expressed higher levels of 3935 and 3623 genes than TAMos from the spleens and the TME, respectively, including the neutrophil hallmark genes *Ly6g*, *Slc27a2* (encoding FATP2), and *S100a9*.^[^ ^31^, ^32^ ^]^ Based on the annotation of the Uniprot database^[^ ^33^ ^]^ and TMHMM Server v2.0 (http://www.cbs.dtu.dk/services/TMHMM/) analysis, we identified 101 and 78 genes encoding cell surface transmembrane proteins that were preferentially expressed in TAMos from the spleens and the TME, respectively (fold change > 10, adjusted *p* value < 0.05 and Fragments Per Kilobase per Million (FPKM) > 0.5) (Figure 7c; Table S1, Supporting Information). Among these genes, 43 were elevated in TAMos from both the spleens and the TME (Figure 7c; Table S1, Supporting Information). Because TAMos derived from the spleens and the TME exert similar effects on T cell differentiation, we subsequently focused mainly on these 43 genes.

To investigate the potential roles of these 43 genes encoding cell surface transmembrane proteins predominantly expressed in TAMos in T_CM_ cell generation, we designed a mitomycin C‐treated transfected HEK293T and OT‐I T cell coculture (MCTOC) screening method, in which OT‐I T cells were incubated with mitomycin C‐treated HEK293T cells overexpressing the indicated transmembrane protein upon T cell activation, followed by evaluation of the proportion of T_CM_ cells in CD8^+^ T cells by flow cytometry (Figure 7d). Employing this system, we have screened 39 of 43 cell surface transmembrane proteins that were successfully expressed in HEK293T cells (Figure S6c, Supporting Information), and found that HEK293T cells ectopically expressing CD300LG, PTPRO, IL6ST, and CD48 substantially promoted T_CM_ cell differentiation, as indicated by an increased percentage of CD44^+^CD62L^+^ cells (Figure 7e,f). These data suggest that at least 4 of the 43 transmembrane proteins could be involved in the induction of T_CM_ cells mediated by TAMos. Furthermore, we also determined that higher CD300LG levels, but not PTPRO, IL6ST or CD48, were associated with better overall survival in cancer patients (Figure S6d, Supporting Information). This observation prompted us to focus on the role of CD300LG in TAMo‐mediated T_CM_ cell polarization. To this end, we used shRNA to knock down CD300LG in TAMos and then cocultured them with CD8^+^ T cells during T cell activation (Figure 7g; Figure S6e, Supporting Information). As shown in Figure 7h and Figure S6f (Supporting Information), TAMos with decreased CD300LG expression levels partially lost their ability to promote T_CM_ cell differentiation, as evident from a decreased proportion of CD44^+^CD62L^+^ cells and downregulated CD62L and CCR7 levels compared with control TAMos. These results demonstrate that surface protein CD300LG in TAMos is important for T_CM_ cell polarization. To further investigate whether CD300LG can directly promote T_CM_ cell generation, we purified the extracellular domain of CD300LG (CD300LG‐EC) and incubated it with OT‐I T cells during T cell activation (Figure 7i). As shown in Figure 7j,k and Figure S6g (Supporting Information), the addition of recombinant CD300LG‐EC led to elevated proportions of T_CM_ cells and increased expression of memory‐associated markers, including TCF‐1, EOMES, IL‐7R, and CD62L. These findings provide evidence that CD300LG, which is highly expressed in TAMos, plays a critical role in the induction of T_CM_ cells.

### 2.8 The Terminally Differentiated CD300LG^high^ACE^low^ TAMo Subset Chiefly Contributes to T_CM_‐Like Cell Development

**Figure 8:**
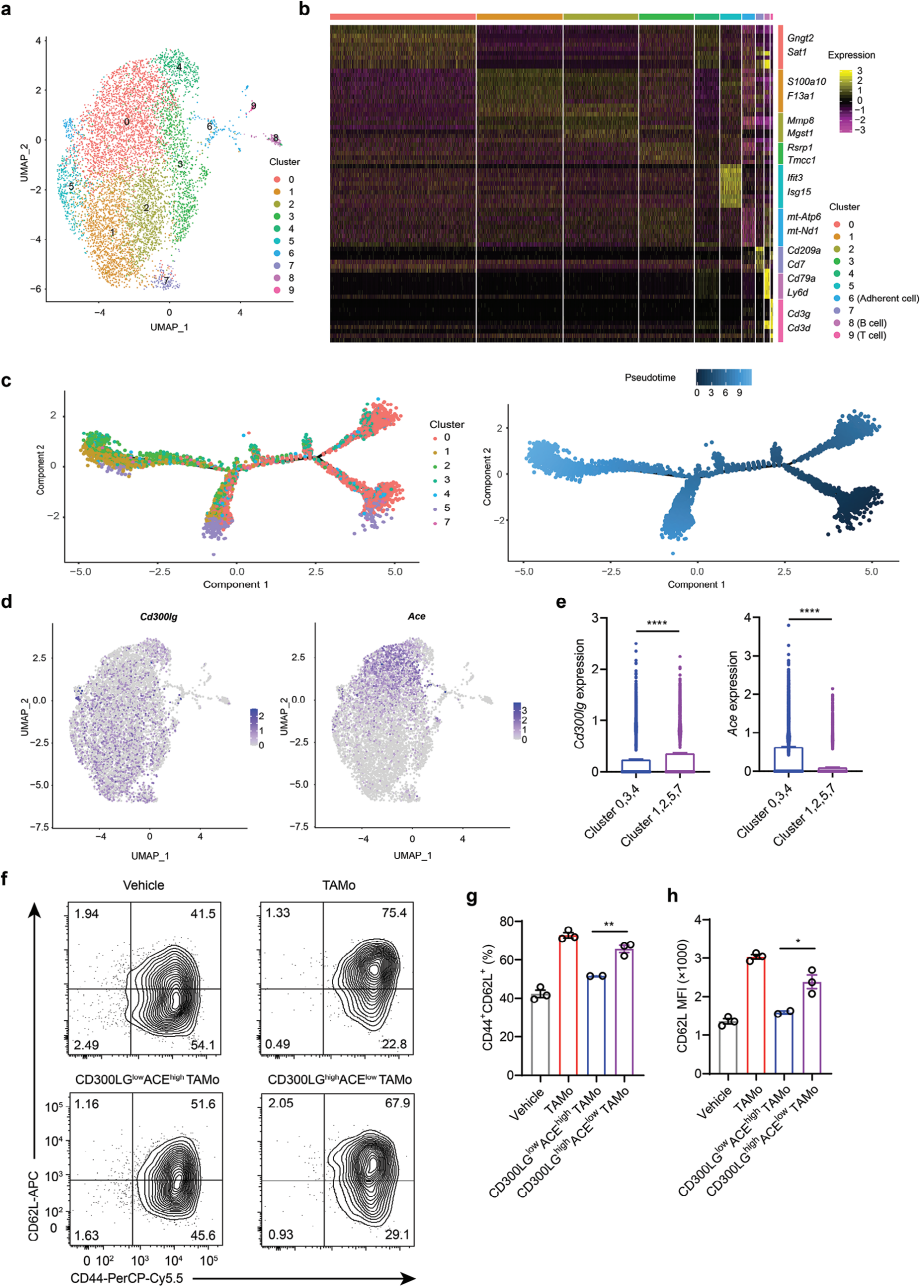
CD300LG^high^ACE^low^ subset in TAMos plays a major role in T_CM_ cell differentiation. ScRNA‐seq analysis of TAMos purified from the spleens of B16‐OVA tumor‐bearing mice (twelve pooled mice, a–e). a) UMAP plot showing ten subclusters of TAMos utilizing graph‐based clustering (*n* = 7113 single cells). b) Heatmap showing scaled expression patterns of the top marker genes in each cell cluster. Clusters 8 and 9 represent B and T cells, respectively. Cluster 6 represents adherent cells. c) Trajectory of cells from the TAMo clusters 0–5 and 7 using the Monocle 2 algorithm. Each dot represents a single cell (*n* = 6788 cells). d) UMAP plots showing expression levels of *Cd300lg* and *Ace* in the different clusters of TAMos. e) *Cd300lg* and *Ace* expression levels in clusters 0, 3, 4 and clusters 1, 2, 5, 7 of TAMos. f–h) CD8^+^ T cells were cocultured with purified TAMos, CD300LG^low^ACE^high^ TAMos, or CD300LG^high^ACE^low^ TAMos derived from B16‐OVA tumor‐bearing mice (five pooled mice) or cultured alone in the presence of anti‐CD3 and anti‐CD28 antibodies, followed by flow cytometry to determine proportions of CD44^+^CD62L^+^ cells f,g) and CD62L levels h) in CD8^+^ T cells (*n* = 2 or 3 cell cultures). Data are representative of two independent experiments f–h) and shown as means ± SEM e,g,h). Statistical significance was assessed using a two‐tailed unpaired Student's *t* test e,g,h). *p < 0.05, ^**^ *p* < 0.01, ^****^ *p* < 0.0001.

To gain insight into cellular mechanisms governing the ability of TAMos to enhance T_CM_ cell differentiation, we conducted 3′ mRNA single‐cell RNA sequencing (scRNA‐seq) analysis on TAMos sorted from the spleens of B16‐OVA tumor‐bearing mice. In total, 7588 cells were analyzed and 27998 gene transcripts were detected. After preprocessing and normalization, 7113 cells of high quality with a median of 2096 gene transcripts detected per cell were used for unbiased graph‐based clustering (Figure S7a, Supporting Information). Cell clusters were visualized using Uniform Manifold Approximation and Projection (UMAP).^[^ ^34^ ^]^ As shown in **Figure** **8**a, ten distinct cell clusters were identified based on the scRNA‐seq profiles. Assessment of canonical marker gene expression revealed that clusters 8 and 9 appeared to be contaminating B and T cells, respectively (Figure 8b; Table S2, Supporting Information). The marker genes of TAMos, including *Ly6c2* (encoding Ly6C) and *Itgam* (encoding CD11b), were expressed in clusters 0–5 and 7, suggesting that TAMos comprise seven distinct subpopulations (Figure S7b, Supporting Information). DEG analysis showed that all other TAMo clusters except cluster 4 possessed upregulated marker genes (Figure 8b; Table S2, Supporting Information). With respect to the top differentially expressed genes, *Gngt2*, *S100a10*, *Mmp8*, *Rsrp1*, *Ifit3*, and *Cd209a* were preferentially expressed in clusters 0, 1, 2, 3, 5, and 7, respectively (Figure 8b). We also analyzed the relationships among the TAMo clusters and pseudotime ordering of cells using the Monocle 2 algorithm.^[^ ^35^ ^]^ As shown in Figure 8c, cluster 0 and clusters 1 and 2 are located at opposite branches of the trajectory. We also noticed that TAMo clusters 1, 2, 5, and 7 at the terminally differentiated stage exhibited higher CD300LG and lower ACE levels, whereas less differentiated TAMo clusters 0, 3, and 4 expressed lower CD300LG and higher ACE levels (Figure 8d,e). Using fluorescence‐activated cell sorting with anti‐ACE antibodies, we separated TAMos into two groups: a less differentiated group (CD300LG^low^ACE^high^) and a terminally differentiated group (CD300LG^high^ACE^low^). We then used a coculture system to explore the roles of the two TAMo subsets on T cell differentiation. As shown in Figure 8f‐h, terminally differentiated TAMos substantially promoted T_CM_ cell generation compared to less differentiated TAMos, as indicated by an increased percentage of CD44^+^CD62L^+^ cells and elevated CD62L levels. These results support the conclusion that the terminally differentiated CD300LG^high^ACE^low^ TAMo subset is the chief contributor to T_CM_ cell differentiation, and also further corroborate a function for CD300LG in TAMo‐mediated T_CM_ cell differentiation.

## 3 Discussion

A comprehensive understanding of tumor‐specific immune characteristics is crucial for generating novel strategies for the treatment of cancer.^[^ ^36^, ^37^ ^]^ In an extensive series of experiments using tumor‐bearing mice and PBMCs from cancer patients, we have identified TAMos as an ACT enhancer that promotes T_CM_ cell differentiation. In contrast to the previously reported immunosuppressive functions of monocytes, which primarily hamper immune responses and promote tumor progression,^[^ ^38^ ^]^ our findings reveal an unexpected beneficial antitumor aspect of this immune cell type, thus providing a deeper understanding of the multiple and complex functions of monocytes.

The TME is usually characterized by hypoxia, low glucose levels, deficient in essential amino acids as well as low pH and elevated lactate levels. This has deleterious effects on the expansion, differentiation, and effector function of T cells, resulting in poor control of tumor growth.^[^ ^39^ ^]^ Nonetheless, recent studies have shown that creating and simulating the inhibitory environment of tumors during T cell culture in vitro could actually improve the clinical efficacy of adoptive immunotherapy. For instance, T cells cultured at pH 6.6 or at high K^+^ concentrations have been found to acquire memory or stem‐like characteristics and exhibit enhanced antitumor activity after transfer into tumor‐bearing mice.^[^ ^18^, ^40^, ^41^ ^]^ In addition, limiting glutamine uptake or maintaining a hypoxic environment in a T cell culture system also enhances T cell persistence in vivo.^[^ ^42^, ^43^ ^]^ In accordance with these previous reports, our findings demonstrate that introducing TAMos into a T cell culture system can augment the proportion of T_CM_ cells and enhance their long‐term persistence in vivo. Using an adoptive transfer model, we showed that tumor‐bearing mice receiving T cells after TAMo exposure exhibited decreased tumor growth accompanied by enhanced antitumor immune responses and lowered T cell exhaustion state. Together, these observations provide additional evidence that pretreatment of T cells with TME‐derived inhibitory factors may be an alternative approach to preserving T cell tolerance to the TME and consequently enhancing ACT efficacy.

In ACT immunotherapy, both the number of transferred T cells and their differentiation state are critical determinants of clinical responses.^[^ ^1^ ^]^ T cells can be expanded through TCR‐mediated stimulation and IL‐2, but this can lead to terminal differentiation into T_EFF_ or T_EX_ cells and consequently, lower therapeutic efficacy. Conversely, the maintenance of a more memory cell‐like state through MEK or WNT inhibition before adoptive T cell transfer is beneficial, whereas the proliferative capacity of tumor‐reactive T cells is limited under these conditions.^[^ ^44^, ^45^ ^]^ In the present study, we determined that the effects of TAMos on T cell proliferation and memory state are uncoupled. T cells modified by TAMo coculture plus L‐NMMA treatment exhibit an equivalent activation signaling and proliferation rate and gain a central memory‐like phenotype. The combination of TAMo coculture and NOS inhibitor treatment represents a feasible way to achieve an optimal balance of T cell proliferation and differentiation in ACT.

TAMos share phenotypic and functional similarities with monocytic myeloid‐derived suppressor cells, and are capable of hampering antitumor immune responses and promoting tumor progression through their ability to transfer NO and methylglyoxal or to secrete TGFβ, IL‐1β and IL‐10 cytokines.^[^ ^46^ ^]^ Consequently, increased TAMos are often associated with poor outcomes in cancer patients.^[^ ^47^ ^]^ Several strategies targeting TAMos are being employed in the pursuit of better tumor control, including inhibition of monocyte recruitment, monocyte depletion, and reprogramming of monocytes.^[^ ^48^ ^]^ We have now shown that TAMos promote T_CM_ cell differentiation via direct physical contact independent of NO secretion. We have also demonstrated the pivotal role of CD300LG, which is highly expressed in TAMos, in T_CM_ cell polarization. Further investigation of CD300LG, a single‐pass transmembrane protein, for its potential clinical relevance appears warranted. Based on the opposing effects of TAMos on T cell proliferation and differentiation demonstrated here, our work highlights the critical need for more precise targeting of the immunosuppressive activities of TAMos in cancer immunotherapy.

When challenged with tumors, many myeloid cells are reprogrammed and differentiated into various abnormal subsets, thus playing complex roles in tumor promotion versus tumor elimination.^[^ ^49^ ^]^ Comprehensive characterization of these cells is therefore crucial for accurate clinical diagnosis and appropriate treatment. Our results show that TAMos exhibit some heterogeneous properties with distinct transcriptional features and differentiation states. Most importantly, we have determined that terminally differentiated CD300LG^high^ACE^low^ monocytes are a major contributor to the generation of T_CM_ cells. In view of the apparent correlation between higher CD300LG levels and a more favorable prognosis in several types of cancer patients, further elucidation of the mechanisms by which CD300LG^high^ TAMos enhance T_CM_ cell differentiation may provide opportunities to improve antitumor immunity by taking advantage of the immuno‐remodeling function of TAMos in different cancer types. Together, our newly identified function of TAMos on T_CM_ cell generation represents a promising avenue of investigation aimed at improving the efficacy and applicability of ACT. It will also be important to further investigate the optimal application of TAMos in combination with other antitumor therapies.

## 4 Experimental Section

### Mice and Cell Lines

Wild‐type C57BL/6J mice were purchased from Vital River Laboratory Animal Technology. OT‐I mice were provided by Dr. Hong Tang (Institute Pasteur of Shanghai Chinese Academy of Sciences). All mice were bred and maintained under specific pathogen‐free conditions. Animal experiments and procedures were conducted in accordance with the Ethics Committee of Peking University Health Science Center (LA2021487). The B16‐F10 and LLC cell lines were purchased from ATCC. The B16‐OVA and LLC‐OVA cell lines were generated by lentiviral infection of the pCCL‐EF1a‐CMV‐NGFR plasmid (from Addgene) encoding OVA protein with a C‐terminal GFP tag into B16‐F10 and LLC cells, respectively.

### Patient Specimens

Peripheral blood samples were obtained from lung cancer patients at the Peking University People's Hospital. All procedures were performed in accordance with the Declaration of Helsinki principles and approved by the Ethics Committee of Peking University Health Science Center (No.2020PHB242‐01). All patients were provided with informed consent before sample collection. None of the patients had received chemotherapy or radiotherapy prior to sample collection.

### Transplantable Tumor Model

Wild‐type C57BL/6J female mice (8–10 weeks old) were unilaterally and subcutaneously inoculated with 1 × 10^6^ B16‐OVA or 2 × 10^6^ LLC tumor cells suspended in 1 × PBS on a shaved flank. Tumor size was measured over time using an electronic caliper, and tumor volume was calculated as length × width^2^/2. Between day 22 and day 28 post‐tumor cell injection, the spleens and tumor tissues of the tumor‐bearing mice were harvested and TAMos, TANs, and TAMs were purified. Mice were euthanized when their tumor volume exceeded 2000 mm^3^.

### Cell Preparation

To isolate leukocytes from murine peripheral lymphoid organs, spleens and lymph nodes were ground in ice‐cold fluorescence‐activated cell sorter (FACS) buffer (PBS containing 1% fetal bovine serum (FBS, Gibco)) and filtered through 40 µm cell strainers, followed by red blood cell lysis using ACK lysis buffer (Solarbio). Isolation of tumor‐infiltrating leukocytes from tumor‐bearing mice was performed as described previously with some modifications.^[^ ^50^ ^]^ Briefly, tumor tissues were mechanically minced and digested with 0.5 mg mL^−1^ collagenase D (Roche) and 0.1 mg mL^−1^ DNase I (Sigma–Aldrich) in RPMI 1640 medium (Gibco) containing 10% FBS at 37 ^°^C for 1 h. Digested tissues were then ground in ice‐cold FACS buffer and passed through 40 µm cell strainers, followed by density gradient centrifugation to enrich viable cells using mouse 1 × lymphocyte separation medium (Dakewe) at 800 × g for 30 min at room temperature. Tumor‐infiltrating leukocytes were then isolated by 40%/80% Percoll (GE Healthcare) gradient centrifugation. After blocking with a monoclonal antibody against CD16/32 (Trustain fcX, Biolegend), splenic and tumor‐infiltrating leukocytes were stained with fluorescence‐tagged antibodies. Mouse TAMos, TANs, and TAMs were purified using an Arial III FACS (BD Biosciences) and then used in subsequent experiments. To isolate CD8^+^ T cells from leukocytes, MagniSort CD8 positive selection beads (eBioscience) were used according to the manufacturer's instructions.

Patient PBMCs were separated from fresh peripheral blood by density gradient centrifugation over a human lymphocyte separation medium (Dakewe). PBMCs were stained with a cocktail of antibodies against CD45, CD11b, and CD14 (see below) or with a panel of antibodies against CD3, CD4, and CD8 (see below). Human TAMos and CD8^+^ T cells were purified from PBMCs using the aforementioned FACS system.

### Flow Cytometry

For surface marker analysis, cells were stained with antibodies in FACS buffer for 30 min at room temperature. For analysis of intracellular cytokines, cells were treated with Protein Transport Inhibitor Cocktail (eBioscience) for 5 h. After surface staining, cells were fixed with IC Fixation Buffer (eBioscience) and permeabilized with 1 × Permeabilization Buffer (eBioscience), followed by staining with antibodies against effector cytokines or molecules. The following fluorescence tagged antibodies were used for flow cytometry: anti‐human CD45 (clone HI30, PE), anti‐mouse/human CD11b (clone M1/70, FITC), anti‐human CD3 (clone OKT3, PerCP‐Cy5.5), anti‐human CD8 (clone HIT8a, FITC), anti‐mouse CD45 (clone 30‐F11, PE‐Cy7), anti‐mouse Ly6C (clone HK1.4, PE), anti‐phospho‐ZAP70/Syk (Tyr319, Tyr352) (clone n3kobu5, PE), anti‐human/mouse TOX (clone TXRX10, EF660), and anti‐mouse CD8 (clone 53–6.7, FITC) (all eBioscience); anti‐human CD14 (clone M5E2, APC), anti‐human CD4 (clone RPA‐T4, APC), anti‐human CD197 (CCR7) (clone G043H7, PerCP‐Cy5.5), anti‐human CD62L (clone DREG‐56, PE), anti‐mouse Ly6G (clone 1A8, PerCP‐Cy5.5), anti‐mouse CD8 (clone 53–6.7, PE/Cyanine7), anti‐mouse CD44 (clone IM7, PerCP‐Cy5.5), anti‐mouse CD44 (clone IM7, APC), anti‐mouse CD62L (clone MEL14, APC), anti‐mouse CD62L (clone MEL14, PE), anti‐mouse TCR Vα2 (clone B20.1, PE), anti‐mouse TCR Vα2 (clone B20.1, FITC), anti‐mouse TNF (clone MP6‐XT22, PE), anti‐mouse IFN‐γ (clone XMG1.2, APC), anti‐mouse CD69 (clone H1.2F3, APC), anti‐mouse CD300LG (clone ZAQ5, PE), and anti‐mouse CD279 (PD‐1) (clone 29F.1A12, PerCP‐Cy5.5) (all BioLegend); anti‐human CD8 (clone OKT8, PerCP‐Cy5.5), and anti‐human CD45RO (clone UCHL1, APC) (Tonbo Biosciences); anti‐mouse ACE (CD143) (clone 230214, Alexa Fluor 647) (R&D); anti‐human/mouse TCF‐1 (clone C63D9, PE) (CST); and anti‐mouse EOMES (clone Dan11mag, AF488) (Thermo Fisher). Antibody‐stained cells were analyzed on a flow cytometer (BD Biosciences) and flow cytometry data were analyzed using FlowJo Software (v10).

### In Vitro Mouse T Cell Activation

OT‐I cells were isolated from the spleens and peripheral (axillary, inguinal, and mesenteric) lymph nodes of 8–12 week old female OT‐I mice. For primary T cell activation, OT‐I cells were cultured alone or cocultured with TANs, TAMos, or TAMs from tumor‐bearing mice at a ratio of 3:1 in round‐bottom 96‐well cell culture plates (Corning) and stimulated with OVA_257‐264_ peptides (2 µg mL^−1^) (Invivogen) for 48 h. Percentages of T_CM_ cells and T cell memory‐associated protein levels in the OT‐I CD8^+^ T cell population were determined by flow cytometry.

To measure anti‐CD3/28 antibody‐mediated T cell activation or recall response, CD8^+^ T cells isolated from the spleens of wild‐type C57BL/6J mice or tumor‐infiltrating leukocytes of tumor‐bearing mice by FACS or MagniSort CD8 positive selection beads were stimulated with plate‐bound anti‐CD3 (2 µg mL^−1^, clone 145‐2C11) (Biolegend), soluble anti‐CD28 (1 µg mL^−1^, clone 37.51) (Biolegend) plus IL‐2 (10 U mL^−1^) (eBioscience, 212‐12) for 2 days in round‐bottom 96‐well cell culture plates. For antigen rechallenge experiments, activated T cells were expanded with 10 U mL^−1^ IL‐2 for 4 days, and reactivated with anti‐CD3 and anti‐CD28 in the presence or absence of TAMos. The levels of markers indicating the memory or exhaustion status of the CD8^+^ T cells were then determined using flow cytometry.

### In Vitro Human T Cell Response

For the experiments shown in Figure 1b, human PBMCs isolated from fresh peripheral blood of lung cancer patients were cultured with plate‐bound anti‐CD3 (1 µg mL^−1^, clone OKT3) (Biolegend), soluble anti‐CD28 (1 µg mL^−1^, clone CD28.2) (Biolegend) and rhIL‐2 (20 ng mL^−1^) (eBioscience, 200–02) for 4 days, followed by evaluation of T cell memory‐associated protein levels and the proportion of monocytes by flow cytometry. For the experiments shown in Figure 4e,f, human CD8^+^ T cells and TAMos were purified from the PBMCs of lung cancer patients. CD8^+^ T cells were then cultured alone or cocultured with human TAMos at a ratio of 2:1 in the presence of anti‐CD3 (1 µg mL^−1^), anti‐CD28 (1 µg mL^−1^) and rhIL‐2 (20 ng mL^−1^) for 4 days followed by the analysis of CD8^+^ T cell subpopulation proportions and CD62L levels by flow cytometry.

### Transwell Experiments

TAMos sorted from the spleens of tumor‐bearing mice were suspended in RPMI 1640 medium and plated on transwell inserts (Corning). The inserts were then cocultured with OT‐I cells that had been plated in the lower compartment of the transwell apparatus at a ratio of 1:3. Both cell populations were treated with OVA_257‐264_ peptides (2 µg mL^−1^) for 48 h, followed by an analysis of CD8^+^ T cell subsets using flow cytometry.

### RNA Isolation and RT‐qPCR Analysis

For RT‐qPCR analysis, cells were washed with PBS, and then RNA was extracted using Trizol reagent (Invitrogen) and reverse transcribed using the GoScriptTM Reverse Transcription System (Promega) according to the manufacturer's instructions. The RT‐qPCR assay was performed on an ABI 7500 system (Applied Biosystems) using the Genious 2 × SYBR Green Fast qPCR Mix (ABclonal). The primers used in this work are listed in Table S3 (Supporting Information).

### Bulk RNA‐Seq

OT‐I cells were cultured alone or cocultured with TAMos or TANs sorted from LLC tumor‐bearing mice in the presence of OVA_257‐264_ peptides (2 µg mL^−1^) for 48 h, followed by purification of CD8^+^ T cells using the FACS system. Splenic or tumor‐infiltrating TANs or TAMos derived from tumor‐bearing mice were sorted using the FACS system. Conventional T_Naïve_, T_CM_, and T_EM_ cells were purified from mouse spleens using FACS system. Total RNA was extracted and quantified using a Nano Photometer spectrophotometer (IMPLEN). Libraries were then prepared from the RNA using a NEBNExt UltraTM RNA Library Prep Kit for Illumina (NEB) according to the manufacturer's instructions and library quality was assessed using the Bioanalyzer 2100 system. Index‐coded libraries were clustered on a cBot Cluster Generation System using a TruSeq PE Cluster Kit v3‐cBot‐HS (Illumina) followed by sequencing on a NovaSeq6000 platform (Illumina) with 150 bp paired‐end reads.

### Analysis of Bulk RNA‐Seq Data

Raw reads in FastQ format were filtered to obtain clean data by removing adaptor sequences and low‐quality reads. Clean reads were then aligned to the mouse mm10 reference genome using Hisat2 (v2.0.5). FeatureCounts (v1.5.0‐p3) were used to count the number of reads mapped to each gene. Based on the length of a gene and the read counts mapped to it, the FPKM of each gene was calculated and used for estimating gene expression levels, followed by DEG analysis using edgeR (v3.18.1). PCA was performed by R function princomp. R package clusterProfiler (v3.16.1) was used for GO enrichment analysis. Gene set enrichment analysis was performed using GSEA software (v4.1.0) and Molecular Signatures Database (v7.5.1). Heatmaps were generated using the R package pheatmap (v1.0.12).

To identify the subsets of CD8^+^ T cells present after coculture with myeloid cells, a logistic regression model using scRNA‐seq data of CD8^+^ T cells including T_Naïve_ cells, T_CM_ cells, T_EM_ cells, T_EFF_ cells, and T_EX_ cells from GSE99254 was constructed.^[^ ^25^ ^]^ First, 100 cells from each subset were randomly choosen and their respective expression matrices were isolated. R package glmnet (v4.0‐2) was used to fit logistic regression models with parameter alpha = 0.5. To evaluate the predictive performance of the models, a ten‐fold cross‐validation was carried out. The offset for each model was calculated as log (*f*/1‐*f*), in which *f* is the fraction of cells in the cluster being trained. After inputting RNA‐seq data into these models as test sets, the predicted logits were converted to probabilities for visualization.^[^ ^51^ ^]^

### In Vitro T Cell‐Mediated Tumor Killing Assay

LLC cells stably overexpressing OVA protein with a GFP tag were incubated with sorted CD8^+^ T cells under different conditions at different effector‐to‐target (E: T) ratios. After staining with 7‐AAD, the percentages of dead cells (7‐AAD^hi^) in GFP positive LLC cells were used for calculating the proportion of T cell‐mediated specific lysis as follows: specific lysis (%) = 100 × (sample lysis (%) – basal lysis (%)) / (100 − basal lysis (%)). Sample lysis (%) reflects the percentage of dead LLC cells in the presence of effector cells at the indicated E:T ratio, and basal lysis (%) reflects the percentage of dead LLC cells in the absence of effector cells.^[^ ^52^ ^]^

### MCTOC Screening Assay

HEK293T cells were transfected with pCCL vectors encoding selected murine surface transmembrane proteins fused with a mCherry tag using jetPRIME transfection reagent (Polyplus). After 36 h, transfected HEK293T cells were treated with mitomycin C (20 µg mL^−1^) (Selleck) for 2 h to inhibit cell proliferation. HEK293T cells ectopically expressing various surface transmembrane proteins were then cocultured with OT‐I T cells and stimulated with anti‐CD3 (2 µg mL^−1^), anti‐CD28 (1 µg mL^−1^) plus IL‐2 (10 U mL^−1^) for 48 h, followed by assessment of T_CM_ cell percentages in OT‐I CD8^+^ T cells by flow cytometry.

### Knockdown of CD300LG or NOS2

ShRNA targeting *Cd300lg* (F: CCGGGCTGTTTCTAGGATCATATATCTCGAGATATATGATCCTAGAAACAGCTTTTTG; R: AATTCAAAAAGCTGTTTCTAGGATCATATATCTCGAGATATATGATCCTAGAAACAGC) or *Nos2* (F: CCGGGAGTTCATCAACCAGTATTATCTCGAGATAATACTGGTTGATGAACTCTTTTTG; R: AATTCAAAAAGAGTTCATCAACCAGTATTATCTCGAGATAATACTGGTTGATGAACTC) was cloned into pLKO.1 plasmids. The lentiviral constructs, psPAX2, and pMD2.G were co‐transfected into HEK293T cells. Viral supernatant was collected and incubated with TAMos derived from the spleens of LLC tumor‐bearing mice. The efficiency of knockdown was determined by RT‐qPCR or flow cytometry assay.

### Adoptive T Cell Therapy

LLC‐OVA cells (2 × 10^6^ per mouse) were injected subcutaneously into the flanks of 6–8 week‐old female NOD‐SCID mice (Charles River) on day 0. At the same time, OT‐I cells were cultured alone or cocultured with TAMos derived from spleens of LLC tumor‐bearing mice in the presence or absence of L‐NMMA (500 µg mL^−1^) (Cayman) under stimulation of OVA_257‐264_ peptides for 48 h. The activated T cells were then expanded with IL‐2 (10 U mL^−1^) for a further four days. Then, T cells (3 × 10^6^ per mouse) were intravenously transferred into LLC‐OVA tumor‐bearing mice on day 7 after tumor inoculation. On the indicated day after T cell transfer, tumor‐bearing mice were euthanized and tumors, spleens, and TDLNs were excised, followed by flow cytometry analysis.^[^ ^53^ ^]^

### NO Measurement

Culture supernatants were collected by centrifugation at 1600 rpm for 5 min, and NO concentrations were determined using a Nitric Oxide Detection Kit (Beyotime), following the manufacturer's instructions.

### CFSE Labeling

For labeling cells with CFSE, CD8^+^ T cells were harvested, washed three times with PBS, and then incubated with CFSE (Biolegend) in the dark for 5 min. The T cells were then washed twice with PBS containing 5% FBS and used for further experiments.

### Protein Purification

A cDNA sequence encoding the extracellular domain of the mouse CD300LG protein together with a FLAG tag at the C terminus was cloned into pCCL plasmid and then transfected into HEK293T cells. Cells were lysed 24 h later in Co‐IP lysis buffer (20 mm Tris‐HCl, pH 8.0, 150 mm NaCl, 1 mm EDTA, 1% NP40, 10% glycerol) freshly supplemented with PMSF and Protease Inhibitor Cocktail (Roche). After centrifugation at 12000 rpm for 30 min, supernatants were incubated with anti‐FLAG M2 beads (Sigma–Aldrich) for 4 h at 4 °C. The protein‐bound beads were washed three times with TBS containing 0.1% (v/v) NP40. Proteins were eluted using 3 × FLAG‐peptide (Sigma–Aldrich).

### Bioinformatics

Correlations between the abundance of monocytes and *SELL*, *CCR7*, or *IL7R* levels were determined with GEPIA2 (http://gepia2.cancer‐pku.cn/#correlation) using the RNA‐seq data in the TCGA database according to the instructions previously reported,^[^ ^54^ ^]^ followed by visualization using R package ggplot2. For survival analysis, the cancer patient cohort was divided into two groups according to the mean value of expression of monocyte signatures genes (*CD14*, *CD33*, *S100A12*), and then HR and Logrank *p* values were determined by GEPIA2.

### ATAC‐Seq and Data Processing

Approximately 50000 viable CD8^+^ T cells sorted from cocultures were used for each library preparation. Cells were lysed in 1 × Lysis Buffer and nuclei were isolated by centrifugation. A TruePrep™ DNA Library Prep Kit V2 for Illumina (Vazyme Biotech) was used to construct transposase‐treated libraries. The mass concentration and molar concentration of the libraries were determined using a Qubit 3.0 Fluorometer and a StepOnePlus™ Real‐Time PCR system, respectively, and the lengths of the inserted fragments were determined using an Agilent HS 2100 Bioanalyzer. Qualified libraries were sequenced using an Illumina HiSeq X ten platform in pair‐end 150 bp style.

Raw data were stored in FastQ format, including the base sequence and corresponding quality information. Trimmomatic (v0.36) was used to remove adaptor‐polluted or low‐quality bases, and reads considered too short (<36 nt) were filtered out to obtain clean data. The clean data were then mapped to the reference mouse genome (mm10) by Bowtie2, and visualized by IGV (Integrative Genomics Viewer). Peaks corresponding to open genomic regions were detected by MACS2, and significantly differential peaks between samples were acquired using MAnorm. The enrichment analysis of the GO term (http://geneontology.org/) was based on a hypergeometric test with a threshold of q < 0.05, to detect significantly enriched pathways.

### scRNA‐Seq and Data Processing

TAMos were sorted from the spleens of B16‐OVA tumor‐bearing mice (pooled samples, *n* = 12). Cells were washed with PBS containing 0.04% bovine serum albumin and resuspended at a final concentration of ≈1000 cells µL^−1^. Single cell suspensions were loaded into the Chromium platform (10X Genomics) to generate gel beads in the emulsion. Libraries were prepared using a Chromium Single Cell 3′ v2 reagent kit (10X Genomics) following the manufacturer's specifications and sequenced on a NovaSeq6000 platform (Illumina).

Sample demultiplexing, alignment to mouse genome mm10, filtering, unique molecular identifier counting, and barcode processing to generate a feature‐barcode (gene‐cell) matrix were performed using the Cell Ranger pipeline v3.0.0 (10X Genomics). A total of 7588 cells that met quality control criteria were obtained with 115082 mean reads per cell (87.1% sequencing saturation).

Secondary analysis of gene expression levels was performed using the Seurat R package (v3.1).^[^ ^55^, ^56^ ^]^ Data were read into the R (v3.6.0) as count matrices and secondary filtration was conducted. Expressed genes detected in more than three cells across the data were retained. Cells expressing fewer than 200 genes or more than 3500 genes were excluded. Cells in which mitochondrially encoded transcripts constituted greater than 5% or in which hemoglobin genes covered more than 16% of transcripts were also excluded, yielding an expression matrix of 7113 cells with 13308 gene transcripts for subsequent analysis.

A total of 13307 variable genes were selected with the “FindVariableFeatures” function of Seurat, followed by PCA. After assessment with the elbow plot approach, the first 20 PCs were selected and dimension reduction by UMAP was performed on these PCs for visualization of the dataset. Unsupervised K‐nearest neighbor clustering with a resolution of 0.6 identified 10 distinct clusters. Marker genes in each cluster were identified using the “FindAllMarkers” function in the Seurat package. Trajectory analysis was performed using the Monocle (v2).^[^ ^35^ ^]^

### Spatial Transcriptomic Analysis

The processed data was downloaded from the Genome Sequence Archive (accession number HRA000437) and analyzed using the R package Seurat. Briefly, the gene‐spot matrix was normalized by the SCTransform function, and dimensionality reduction was performed by the RunPCA function. To determine the cell abundance score in each spot, the AddModuleScore function to add the specific cell signature genes as a feature and then the SpatialFeaturePlot function to visualize results was used.

### Statistical Analysis

Statistical analyses were performed using GraphPad Prism (v8.0.2) or RStudio (v1.2.5042). A two‐tailed unpaired Student's *t*‐test or a two‐way ANOVA test was used to calculate differences between the two groups. Log‐rank tests were used to analyze survival curves. Differences were considered as statistically significant at ^*^ *p* < 0.05, ^**^ *p* < 0.01, ^***^ *p* < 0.001, and ^****^ *p* < 0.0001. Unless otherwise specified, the data shown are means ± SEM. No blinding was used.

## Conflict of Interest

The authors declare no conflict of interest.

## Author Contributions

Z.Y., L.L., and Y.Y. conceived the study. Z.Y. and L.L. designed the experiments and interpreted the results. Z.Y. and L.L. performed most of the experiments. Z.Z. and Z.H. assisted in the experiments. Z.Y. performed bioinformatics analyses on public scRNA‐seq and TCGA databases. B.L., Yuan J., and Y.D. contributed to flow cytometry experiments. J.G. assisted with the establishment of tumor mouse models. Yan J., J.L., and G.W. provided technical assistance. Z.Y., L.L., and Y.Y. wrote the paper. Y.Y. supervised biological research and the study design.

## Supporting information

Supporting Information

Supplemental Table 1

Supplemental Table 2

Supplemental Table 3

## Data Availability

The data that support the findings of this study are available from the corresponding author upon reasonable request.
