# Supplementary material for: Tumor‐Associated Monocytes Reprogram CD8+ T Cells into Central Memory‐Like Cells with Potent Antitumor Effects
Source: Adv Sci (Weinh). 2024 Feb 22;11(16):2304501. doi: 10.1002/advs.202304501 (PMC11040375; doi:10.1002/advs.202304501)
Supplement: Supplementary file 1 — Supporting Information [file ADVS-11-2304501-s003.pdf]

ADVANCED SCIENCE
Open Access

Supporting Information

for *Adv. Sci.*, DOI 10.1002/advs.202304501

Tumor-Associated Monocytes Reprogram CD8$^+$ T Cells into Central Memory-Like Cells with Potent Antitumor Effects

*Zeliang Yang, Liang Liu, Zhenyu Zhu, Zixi Hu, Bowen Liu, Jingjing Gong, Yuan Jin, Juan Luo, Yichen Deng, Yan Jin, Guangxi Wang and Yuxin Yin**

Supporting Information

Tumor-associated Monocytes Reprogram CD8$^+$ T Cells into Central Memory-like Cells with Potent Antitumor Effects

*Zeliang Yang, Liang Liu, Zhenyu Zhu, Zixi Hu, Bowen Liu, Jingjing Gong, Yuan Jin, Juan Luo, Yichen Deng, Yan Jin, Guangxi Wang and Yuxin Yin**

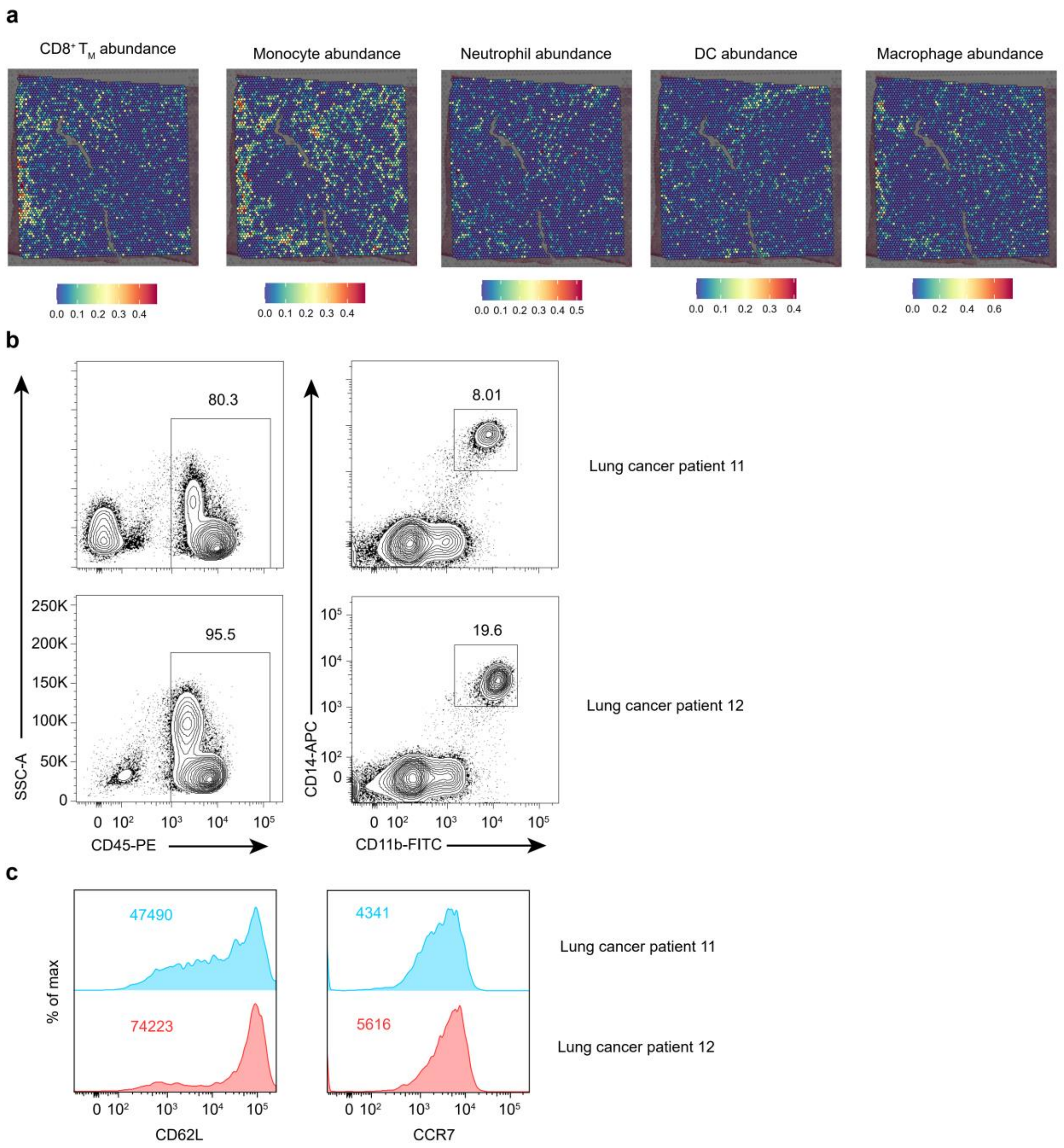

Figure S1. CD14^{+} monocytes show positive correlation with T_M cells

a, Spatial feature plots of different cell signatures on hepatocellular carcinoma (HCC). **b**, Representative plots showing gating strategy and proportions of monocytes in untreated PBMCs from lung cancer patients in Figure 1b. **c**, Representative plots displaying expression levels of the memory markers CD62L and CCR7 in CD8^{+} T cells from stimulated PBMCs of lung cancer patients in Figure 1b.

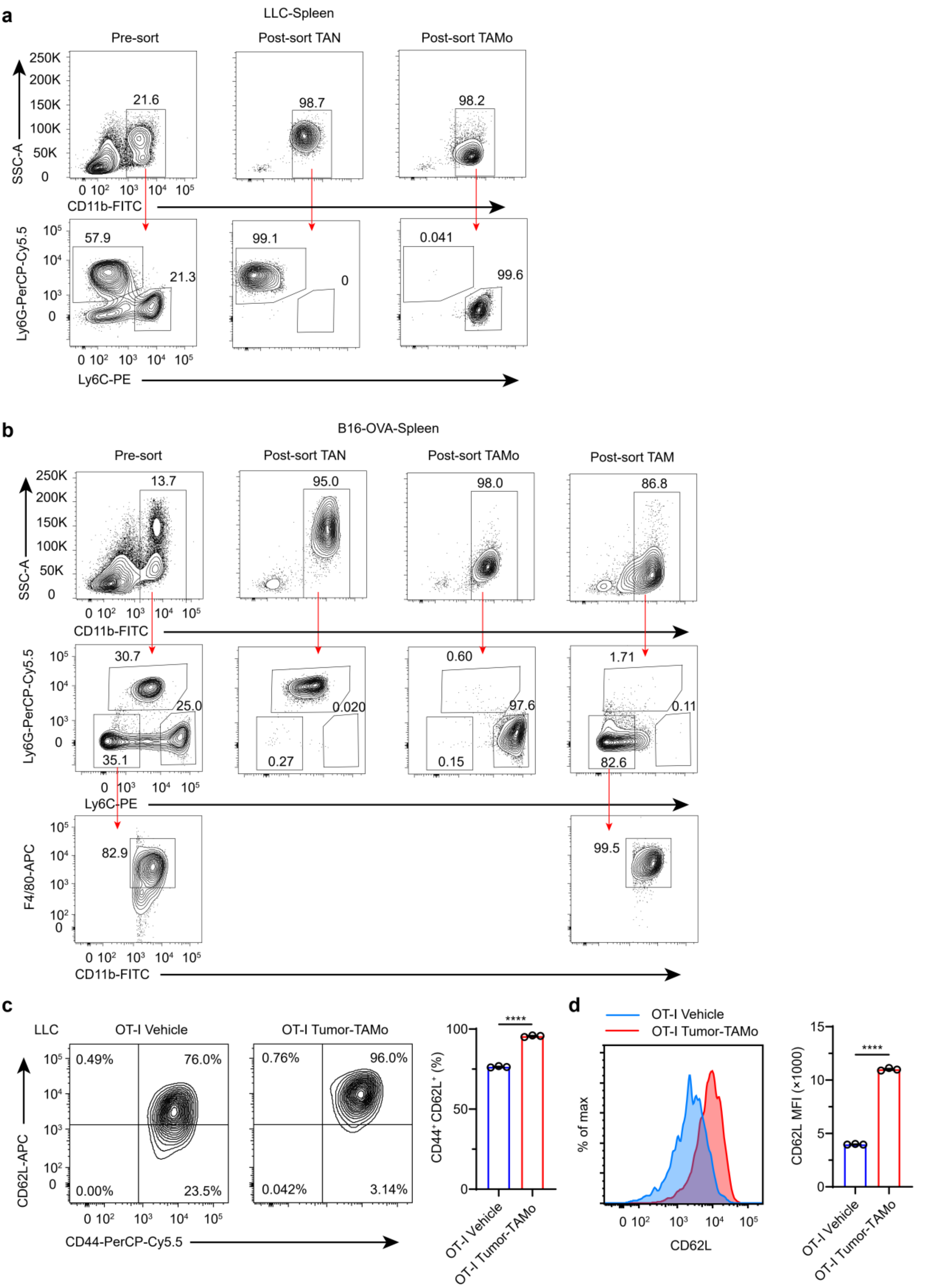

Figure S2. TME-derived TAMos potentiate central memory-like phenotype

a, Representative plots of pre-sort or post-sort TAMos or TANs from the spleens of LLC tumor-bearing mice. **b**, Representative plots of pre-sort or post-sort TAMos, TANs or TAMs

from the spleens of B16-OVA tumor-bearing mice. **c**, **d**, OT-I cells were co-cultured with TAMos sorted from the TME of LLC tumor-bearing mice and activated with $OVA_{257-264}$ peptides for 48 hr. The proportions of $CD44^{+}CD62L^{+}$ cells (**c**) and CD62L expression levels (**d**) in OT-I $CD8^{+}$ T cells were determined by flow cytometry (10 pooled mice, $n = 3$ cell cultures). Data are representative of three independent experiments and shown as means ± SEM (**c**, **d**). Statistical significance was assessed using a two-tailed unpaired Student's *t* test (**c**, **d**). **** $P < 0.0001$.

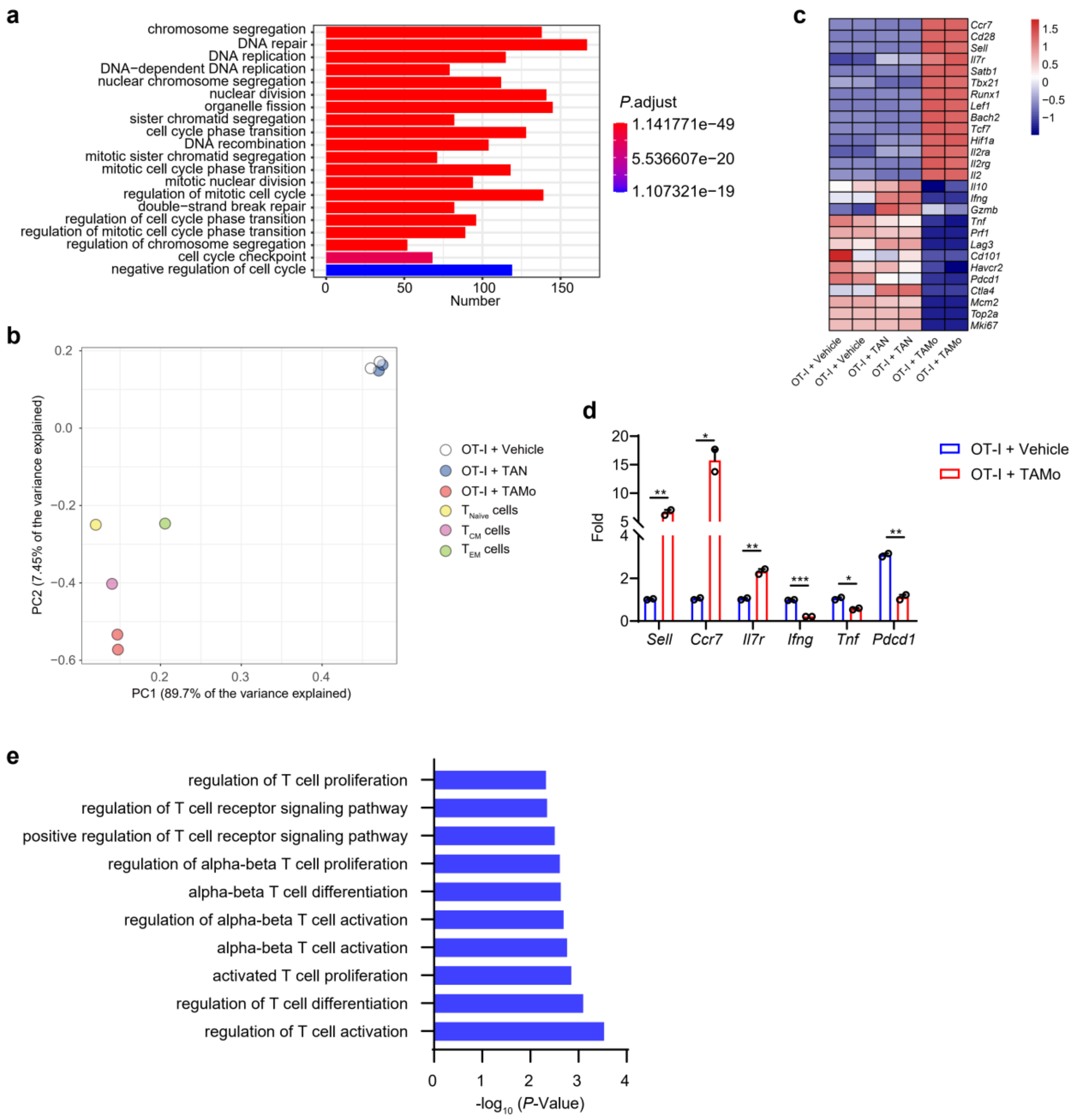

Figure S3. Transcriptional and epigenetic analysis of T cells after exposure to vehicle or TAMos

a, The downregulated genes in TAMo-exposed T cells versus vehicle-treated T cells were analyzed using the GO database. **b**, Principal components analysis of transcriptomes of OT-I cells cultured alone or co-cultured with TANs or TAMos and conventional $T_{Naïve}$, T_{CM}, T_{EM} cells from mouse spleens. **c**, Heatmap showing differentially expressed genes (DEGs) of OT-I $CD8^+$ T cells under different culture conditions. **d**, $CD8^+$ T cells were sorted from co-culture systems in which OT-I cells were treated with TAMos or untreated under $OVA_{257-264}$ stimulation, followed by RT-qPCR analysis (n = 2 cell cultures). **e**, GO enrichment analysis of more accessible T cell-related genes in T cells co-cultured with TAMos compared with T cells cultured alone. Data are representative of three independent experiments and shown as

means ± SEM (**d**). Statistical significance was assessed using a two-tailed unpaired Student's *t* test (**d**). * $P < 0.05$, ** $P < 0.01$, *** $P < 0.001$.

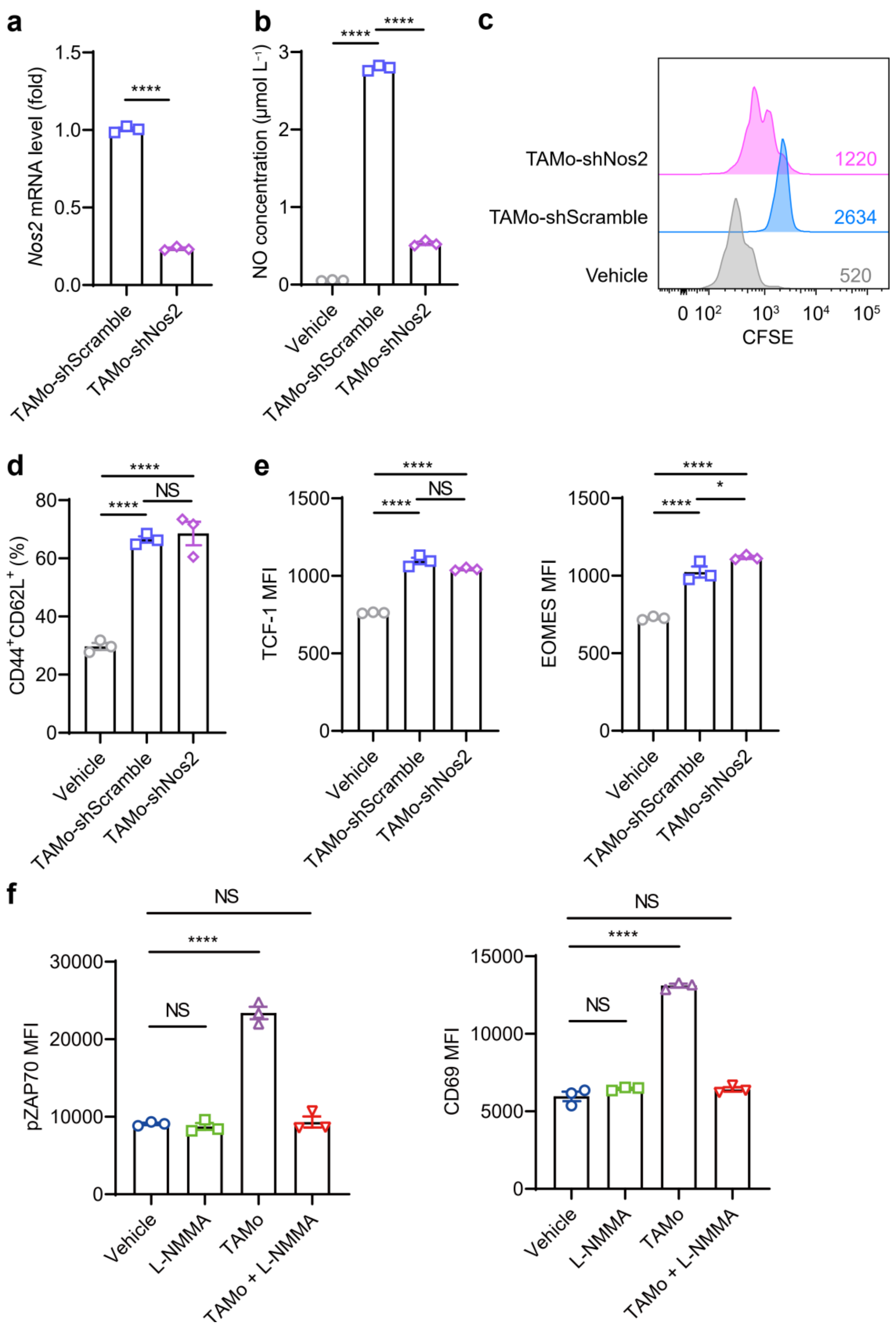

Figure S4. ***Nos2*-deficient TAMos exhibit similar ability to promote T_{CM} cell production compared with control TAMos**

a, RT-qPCR analysis showing *Nos2* mRNA levels in TAMos expressing shRNA targeting *Nos2* or scramble shRNA (*n* = 3 replicates). **b**, NO production in supernatants of CD8$^+$ T cells

activated with anti-CD3 and anti-CD28 antibodies alone, or in the presence of TAMos expressing shRNA targeting *Nos2* or scramble shRNA (n = 3 cell cultures). **c**, CFSE-labelled $CD8^+$ T cells were cultured alone or co-cultured with TAMos expressing shRNA targeting *Nos2* or scramble shRNA under stimulation of anti-CD3 and anti-CD28 antibodies for 48 hr, and T cell proliferation was determined by mean fluorescence intensity of CFSE. **d, e**, Flow cytometry analysis showing the percentages of $CD44^+CD62L^+$ cells (**d**, n = 3 cell cultures) and the expression levels of TCF-1 and EOMES (**e**, n = 3 cell cultures) in the $CD8^+$ T cells cultured alone or co-cultured with TAMos expressing shRNA targeting *Nos2* or scramble shRNA for 48 hr upon T cell activation. **f**, $CD8^+$ T cells were cultured alone or co-cultured with TAMos derived from the spleens of LLC tumor-bearing mice in the presence or absence of L-NMMA for 48 hr under stimulation of anti-CD3 and anti-CD28 antibodies, followed by flow cytometry to determine phosphorylated ZAP70 and CD69 levels (n = 3 cell cultures). Data are shown as means ± SEM. Statistical significance was assessed using a two-tailed unpaired Student's t test. *$P < 0.05$, **** $P < 0.0001$. NS, not significant.

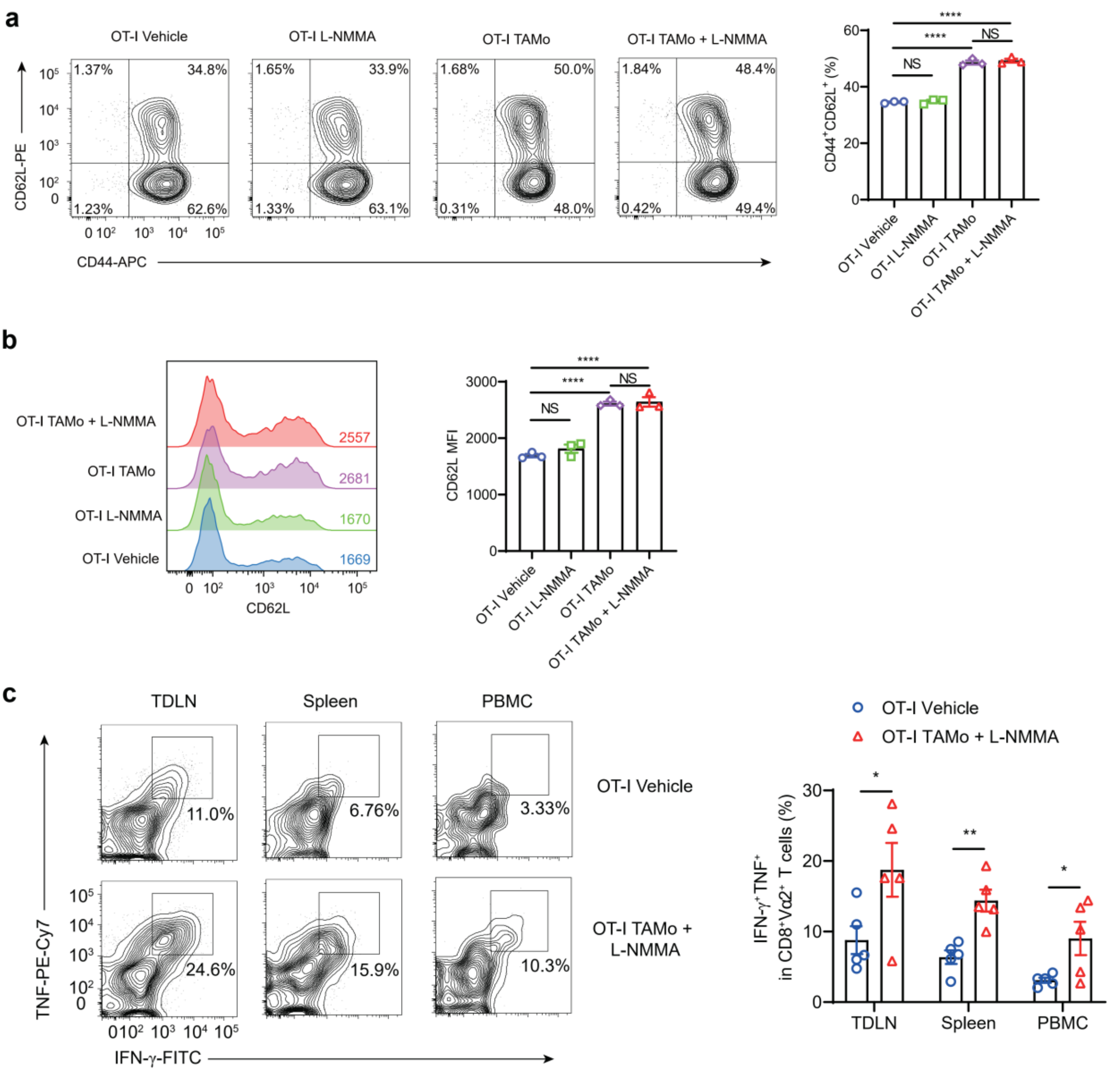

Figure S5. TAMo-exposed T cells exhibit higher proportions of T_{CM} cells after IL-2 expansion and TAMos under L-NMMA treatment endow T cells with enhanced effector function *in vivo*

a, **b**, OT-I cells were cultured alone or co-cultured with TAMos in the presence or absence of L-NMMA and stimulated with $OVA_{257\text{-}264}$ peptides for 48 hr. Enriched $CD8^+$ T cells were then expanded for another 4 days using IL-2. **a**, The percentages of T_{CM} cells in $CD8^+$ T cells were determined by flow cytometry (*n* = 3 cell cultures). **b**, Flow cytometry analysis of CD62L expression levels in $CD8^+$ T cells (*n* = 3 cell cultures). **c**, Flow cytometry analysis showing IFN-γ and TNF expression levels in transferred $CD8^+V\alpha2^+$ OT-I cells in different organs from mice receiving vehicle-treated T cells or L-NMMA-treated TAMo-exposed T cells (*n* = 5 mice per group). Data are shown as means ± SEM. Statistical significance was assessed by a two-tailed unpaired Student's *t* test. *$P < 0.05$, ** $P < 0.01$, and **** $P < 0.0001$. NS, not significant.

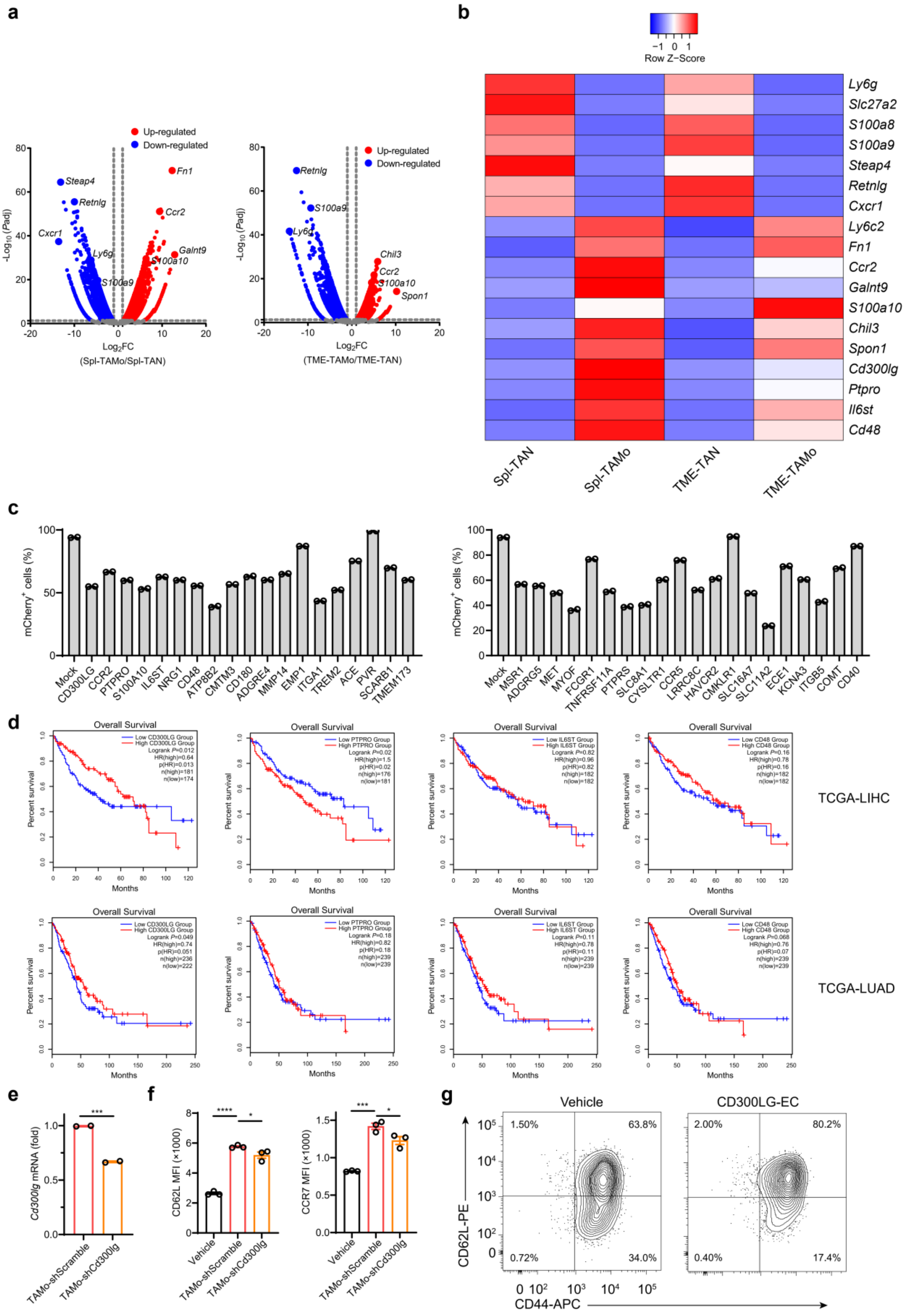

Figure S6. Identification of CD300LG highly expressed in TAMos and validation of its role in T_{CM} cell differentiation

a, Volcano plots summarizing RNA-seq profiling that indicates differentially expressed genes in TANs and TAMos sorted from the spleens of B16-OVA tumor-bearing mice (15 pooled mice) or from the TME of LLC tumor-bearing mice (10 pooled mice). FC, fold change. **b**, Heatmap displaying the top differentially expressed genes in TANs and TAMos from the spleens of B16-OVA tumor-bearing mice (15 pooled mice) or the TME of LLC tumor-bearing mice (10 pooled mice). **c**, Flow cytometry analysis of the proportions of HEK293T cells expressing mCherry-tagged mock or surface transmembrane proteins after transfection with the corresponding vectors (n = 2 cell cultures). **d**, The relationship of overall survival of patients with LIHC (n = 364 samples) or LUAD (n = 478 samples) and expression levels of CD300LG, PTPRO, IL6ST or CD48. **e**, *Cd300lg* mRNA levels in TAMos infected with lentivirus encoding shRNA targeting *Cd300lg* or scramble shRNA (n = 2 cell cultures). **f**, CD62L and CCR7 levels in $CD8^+$ T cells co-cultured with TAMos infected with lentivirus encoding shRNA targeting *Cd300lg* or scramble shRNA (n = 3 cell cultures). **g**, Representatives of flow cytometry plot in Figure 7j. Data are representative of two independent experiments (**c**, **e**, **f**) and shown as means ± SEM (**c**, **e**, **f**). Statistical significance was assessed by log-rank (Mantel-Cox) test (**d**) or two-tailed unpaired Student's t test (**e**, **f**). *$P < 0.05$, *** $P < 0.001$, and **** $P < 0.0001$.

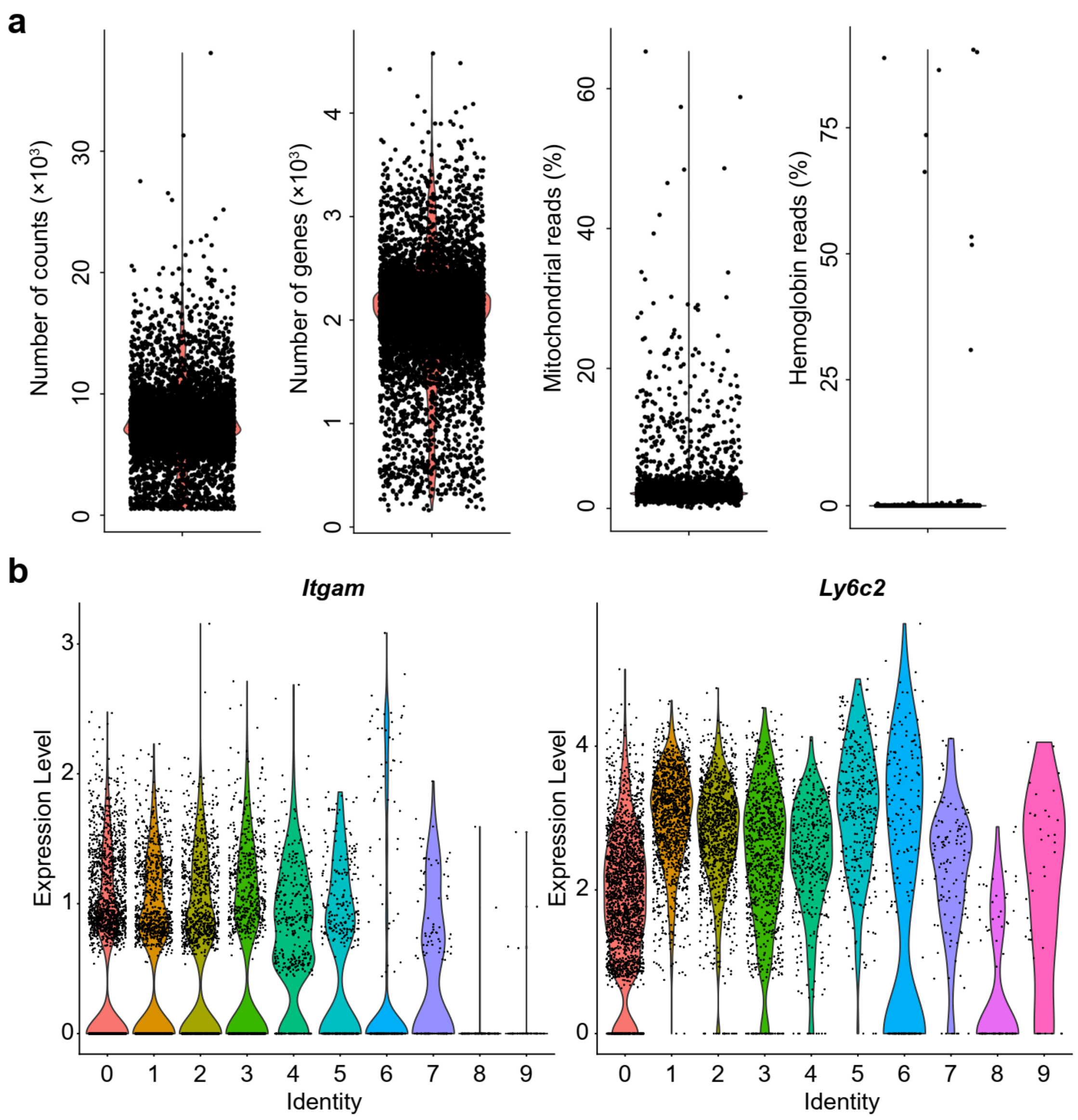

Figure S7. ScRNA-seq analysis of TAMos

a, Violin plots displaying the distribution of the number of counts, number of genes, percentage of mitochondrial reads and percentage of hemoglobin reads in the pre-filtered scRNA-seq data of TAMos sorted from the spleens of B16-OVA tumor-bearing mice. Each dot represents an individual cell (12 pooled mice, n = 7,588 cells). **b**, Violin plots of expression levels of the TAMo marker genes *Itgam* and *Ly6c2* in the clusters of sorted TAMos.
